# Minimal SPI1-T3SS effector requirement for *Salmonella* enterocyte invasion and intracellular proliferation *in vivo*

**DOI:** 10.1371/journal.ppat.1006925

**Published:** 2018-03-09

**Authors:** Kaiyi Zhang, Ambre Riba, Monika Nietschke, Natalia Torow, Urska Repnik, Andreas Pütz, Marcus Fulde, Aline Dupont, Michael Hensel, Mathias Hornef

**Affiliations:** 1 Institute of Medical Microbiology, RWTH University Hospital, Aachen, Germany; 2 Division of Microbiology, University of Osnabrück, Osnabrück, Germany; 3 Department of Biosciences, University of Oslo, Oslo, Norway; 4 Institute of Microbiology and Epizootics, Freie Universität Berlin, Berlin, Germany; University of California Davis School of Medicine, UNITED STATES

## Abstract

Effector molecules translocated by the *Salmonella* pathogenicity island (SPI)1-encoded type 3 secretion system (T3SS) critically contribute to the pathogenesis of human *Salmonella* infection. They facilitate internalization by non-phagocytic enterocytes rendering the intestinal epithelium an entry site for infection. Their function *in vivo* has remained ill-defined due to the lack of a suitable animal model that allows visualization of intraepithelial *Salmonella*. Here, we took advantage of our novel neonatal mouse model and analyzed various bacterial mutants and reporter strains as well as gene deficient mice. Our results demonstrate the critical but redundant role of SopE_2_ and SipA for enterocyte invasion, prerequisite for transcriptional stimulation and mucosal translocation *in vivo*. In contrast, the generation of a replicative intraepithelial endosomal compartment required the cooperative action of SipA and SopE_2_ or SipA and SopB but was independent of SopA or host MyD88 signaling. Intraepithelial growth had no critical influence on systemic spread. Our results define the role of SPI1-T3SS effector molecules during enterocyte invasion and intraepithelial proliferation *in vivo* providing novel insight in the early course of *Salmonella* infection.

## Author summary

Non-typhoidal *Salmonella* represent a major causative agent of gastroenteritis worldwide. Hallmark of the pathogenesis is their ability to actively invade the intestinal epithelium by virtue of their type 3 secretion system that delivers bacterial virulence factors directly into the host cell cytosol. The role of these virulence factors during enterocyte entry and intraepithelial growth has only been investigated *in vitro* since the previously established *in vivo* models in small animals did not allow visualization of intraepithelial *Salmonella*. However, immortalized cell lines lack the overlaying mucus layer, final cell lineage differentiation, apical-basolateral polarization as well as continuous migration along the crypt villus axis and thus the role of virulence factors during the *Salmonella* infection *in vivo* has remained largely undefined. Here, we took advantage of our novel neonatal mouse infection model and for the first time systematically analyzed the importance of *Salmonella* virulence factors for enterocyte invasion and intraepithelial growth.

## Introduction

Non-typhoidal *Salmonella* like *Salmonella enterica* subsp. *enterica* sv. Typhimurium (*S*. Typhimurium) represent a major causative agent of gastroenteritis in humans worldwide [1, 2]. Infection usually occurs through the ingestion of contaminated food or drinking water. *Salmonella* colonizes the distal small intestine, penetrates the intestinal epithelium and induces a strong inflammatory tissue response provoking the main clinical symptoms such as abdominal pain and diarrhea. In the healthy human host, non-typhoidal *Salmonella* remain restricted to the intestinal tissue. In contrast, spread to systemic organs associated with high mortality is observed in immunosuppressed individuals and newborns. This renders *Salmonella* one of the most important causative agents of neonatal sepsis and meningitis in parts of Asia and sub-Saharan Africa [3, 4]

*Salmonella* employs a multitude of virulence factors to overcome the mucosal barrier and evade the cellular and humoral host defence [5]. Effector molecules secreted by the *Salmonella* pathogenicity island (SPI)1-type 3 secretion system (T3SS) act during the early phase of infection and enable *Salmonella* to penetrate the intact intestinal epithelial barrier and reach the subepithelial tissue [6, 7]. SPI1-T3SS effector molecules such as SipA, SopA, SopB, and SopE_2_ intimately interact with host cell processes and manipulate cellular functions such as F-actin dynamics, signal transduction, chemokine secretion, fluid homeostasis, membrane trafficking and tight junction formation [6, 8–24]. Thereby, they facilitate enterocyte invasion followed by intraepithelial proliferation, histological hallmark of *Salmonella* pathogenesis [7, 25–29]. An intact SPI1-T3SS has been strongly associated with mucosal inflammation and diarrhea and thus the clinical symptoms of *Salmonella* gastrointestinal infection in different models [30–36]. The presence in all *Salmonella* subspecies and clinical isolates indicates a critical and non-redundant function of SPI1 also during human infection [37, 38].

The functional relevance of the SPI1-T3SS for tissue infiltration, mucosal inflammation and enhanced fluid secretion *in vivo* has first been characterized using the bovine ileal loop or oral calf infection model [13, 32–34, 39–42]. Infection of calves mimics the disease in humans, characterized by small intestinal mucosal inflammation with chemokine secretion and leukocyte infiltration as well as enhanced fluid secretion [43–45]. Epithelial invasion has also been observed in guinea pig, swine and rabbit intestinal tissue [25–27, 46, 47]. In the most widely used animal model of adult mice, however, oral administration of *Salmonella* does not lead to detectable epithelial invasion and mucosal inflammation but causes a typhoid fever-like systemic infection following a largely SPI1-independent M cell-mediated mucosal translocation [48–50]. Streptomycin administration prior to infection facilitates bacterial expansion and leads to mucosal inflammation [35]. *Salmonella*-induced tissue pathology, however, is largely restricted to the caecum and colon and SPI1-dependent epithelial invasion is observed at low frequency due to rapid enterocyte exfoliation [28, 29, 35, 51, 52]. Although enterocyte invasion and intracellular proliferation has been observed in epithelial cells of the caecum of *S*. Typhimurium infected adult animals and mechanisms of host defence have been investigated [29, 53], the role of individual SPI1-T3SS mediated effector molecules during the early steps of *Salmonella* infection *in vivo* has remained ill-defined.

We have recently introduced a novel oral *Salmonella* infection model using neonate mice [50]. In this model, *Salmonella* invades the neonatal small intestinal epithelium, forms intraepithelial microcolonies and spreads to systemic organs in a strongly SPI1-T3SS dependent manner. Here, we used this model and employed wild type *Salmonella*, *sopABE*_*2*_*sipA* quadruple mutants complemented with individual effector molecules, the respective triple mutants, s*opE*_*2*_*sipA*, *sopAE*_*2*_, and *sopBE*_*2*_ double mutants as well as *sipA*, *sopB*, and *sopE*_*2*_ single mutants in combination with wild type and myeloid differentiation primary response gene 88 (MyD88)^-/-^ mice to investigate the contribution of individual SPI1-T3SS effector molecules and host factors during epithelial cell invasion and intraepithelial microcolony formation *in vivo*.

## Results

### Comparative analysis of individual SPI1-T3SS effector molecules by complementation

We initially employed a previously established approach using a *sopABE*_*2*_*sipA* quadruple mutant *S*. Typhimurium strain complemented *in trans* by an expression plasmid encoding the respective SPI1 virulence factors SipA, SopA, SopB or SopE_2_ [42]. Consistent with our previous results obtained using the SPI1-T3SS defective *S*. Typhimurium *invC* mutant, the *sopABE*_*2*_*sipA* quadruple mutant was unable to invade murine enterocytes, failed to induce a significant transcriptional epithelial response and remained restricted to the gut lumen (S1A–S1F Fig) [50]. Complementation with *SipA* alone significantly enhanced *S*. Typhimurium viable counts in isolated enterocytes, spleen, liver and mesenteric lymph node (MLN) tissue and induced transcriptional epithelial activation (S1A–S1F Fig). Consistently, immunostaining visualized *sipA* complemented Δ*sopABE*_*2*_*sipA Salmonella* within intestinal epithelial cells (S1G Fig). Also *sopE*_*2*_ complemented quadruple mutant *Salmonella* were observed intracellularly (S1G Fig). In contrast, no enterocyte invasion could be observed for *sopA* or *sopB* complemented Δ*sopABE*_*2*_*sipA Salmonella* (S1G Fig). Whereas SopE_2_ is conserved in all pathogenic strains of *Salmonella*, some strains additionally harbor a homologue broad-spectrum guanine nucleotide exchange factor, SopE [54–56]. Also SopE enhanced the invasive behavior leading to a significant increase in enterocyte invasion, transcriptional stimulation as well as spread to systemic tissues (S2A–S2F Fig) [57]. Importantly, albeit able to invade the epithelium, *sipA*, *sopE*_*2*_ and *sopE* complemented Δ*sopABE*_*2*_*sipA Salmonella* were unable to proliferate and generate intraepithelial microcolonies (S1G Fig, S2G Fig).

### Analysis of enterocyte invasion using triple mutants

**Fig 1:**
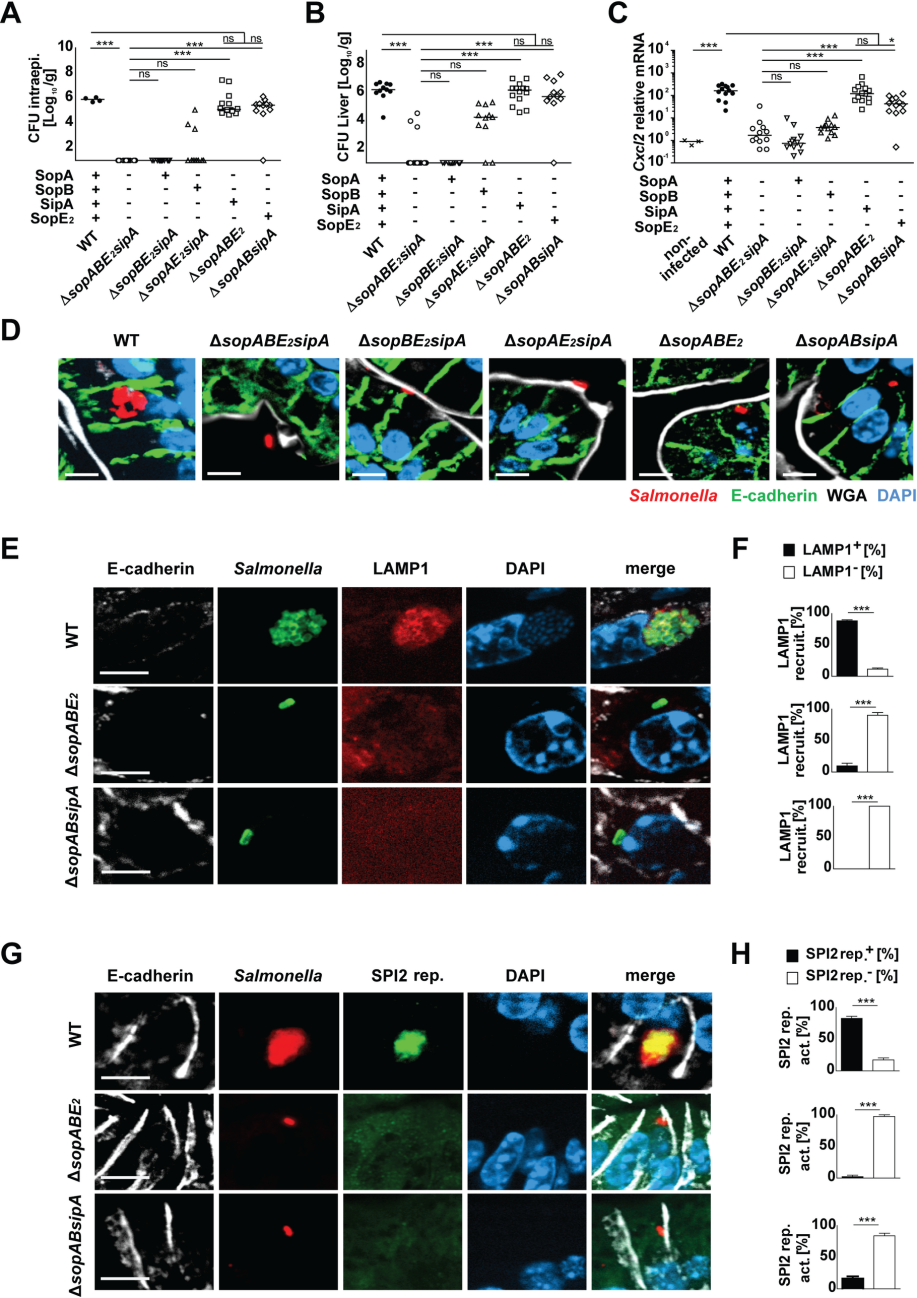
Comparative analysis of the role of SPI1-T3SS effector molecules SopA, SopB, SipA, and SopE_2_ using triple mutants. 1-day-old C57BL/6 mice were orally infected with 100 CFU wild type (WT) (filled circles), isogenic quadruple *sopABE*_*2*_*sipA* mutant (open circles), or Δ*sopBE*_*2*_*sipA* (open inverted triangles), Δ*sopAE*_*2*_*sipA* (open triangles), Δ*sopABE*_*2*_ (open squares), or Δ*sopABsipA* (open diamonds) *S*. Typhimurium. Viable counts in **(A)** isolated gentamicin-treated enterocytes and **(B)** total liver tissue homogenate at 4 days post infection (p.i.). **(C)** Quantitative RT-PCR for *Cxcl2* mRNA in total RNA prepared from enterocytes isolated at 4 days p.i.. Values were normalized to uninfected age-matched control animals (crosses). Individual values and the mean from at least two independent experiments are shown (n = 4–6 animals per group). **(D)** Immunostaining for *Salmonella* (red) in small intestinal tissue sections at 4 days p.i. with 100 CFU WT *S*. Typhimurium (WT), Δ*sopABE*_*2*_*sipA* quadruple mutant, or Δ*sopBE*_*2*_*sipA*, Δ*sopAE*_*2*_*sipA*, Δ*sopABE*_*2*_, or Δ*sopABsipA* triple mutant bacteria. Counterstaining with E-cadherin (green), WGA (white) and DAPI (blue). Bar, 5 μm. **(E)** Co-immunostaining for *Salmonella* WT, Δ*sopABE*_*2*_, or Δ*sopABsipA* (green) and LAMP1 (red) in small intestinal tissue sections at day 4 p.i.. Counterstaining with E-cadherin (white) and DAPI (blue). Bar, 5 **μm**. **(F)** Quantitative evaluation of the percentage of intraepithelial *S*. Typhimurium associated with LAMP1 staining. Three tissue sections per infected neonate (n = 3) were analyzed at day 4 p.i.. Results represent the mean ± SD. **(G)** Co-immumostaining for *Salmonella* WT, Δ*sopABE*_*2*_ or Δ*sopABsipA* (red) and the GFP-expressing SPI2 reporter (pM973; green) in small intestinal tissue sections at day 4 p.i.. Counterstaining with E-cadherin (white) and DAPI (blue). Bar, 5μm. **(H)** Quantitative analysis of the percentage of intraepithelial *S*. Typhimurium expressing the SPI2 reporter. All microcolonies from three tissue sections per infected neonate were analyzed (n = 3) at day 4 p.i.. Results represent the mean ± SD.

To overcome the technical obstacles associated with *trans*-complementation such as plasmid loss, multiple effector gene copies or an impaired access of regulatory elements we next employed triple mutants leaving the fourth SPI1-T3SS effector gene under the native regulatory control. In accordance with our previous results, *sopABE*_*2*_ and *sopABsipA* mutant *Salmonella* expressing solely a functional SipA and SopE_2_ effector, respectively, displayed the ability to invade primary enterocytes and enhance *Cxcl2* and *Cxcl5* mRNA expression in the intestinal epithelium (Fig 1A and 1C, S3C Fig). Both mutants also penetrated the mucosal barrier reaching liver, MLN, and spleen tissue at numbers comparable to wild type bacteria (Fig 1B, S3A and S3B Fig). In contrast, *sopBE*_*2*_*sipA and sopAE*_*2*_*sipA* mutant bacteria expressing *sopA* or *sopB* alone failed to significantly invade and penetrate the epithelium, stimulate a transcriptional response and spread to systemic organs (Fig 1A–1C). Consistently, immunostaining identified intraepithelial *Salmonella* in the presence of SipA or SopE_2_ but not SopA or SopB (Fig 1D). Again, despite the intraepithelial localization, SipA expressing Δ*sopABE*_*2*_ or SopE_2_ expressing Δ*sopABsipA Samonella* failed to proliferate intracellularly.

Following internalization, *Salmonella* manipulates maturation of the endosomal compartment. This recruits the lysosomal-associated membrane protein (LAMP)1 from Golgi-derived vesicles [58] and provides the environmental signals that coordinate the expression of SPI2-T3SS effector genes and the development of a replicative compartment [59, 60]. LAMP1 recruitment to intraepithelial bacteria was observed following infection with wild type but not Δ*sopABE*_*2*_ or Δ*sopABsipA Salmonella* (Fig 1E and 1F). Additionally, we used a previously described SPI2 reporter construct expressing *gfp* under the control of the *ssaG* promoter to analyze the induction of SPI2-encoded genes [52]. SPI2 reporter activity was detected in intraepithelial wild type but not SipA expressing Δ*sopABE*_*2*_ or SopE_2_ expressing Δ*sopABsipA Salmonella* (Fig 1G and 1H).

Together, these results identifed the critical but redundant role of SopE_2_ and SipA for enterocyte invasion *in vivo* and highlighted the requirement of enterocyte invasion for transcriptional stimulation. Notably, invasion *per se* appeared not to be sufficient to generate an appropriate intracellular niche allowing intraepithelial bacterial proliferation. On the other hand, the formation of intraepithelial microcolonies did not significantly promote systemic spread. The ability and degree of individual SPI1-T3SS effector molecules to confer an enterocyte-invasive phenotype differed markedly between the situation *in vivo* and a classical *in vitro* cell culture-based invasion assay (S1H Fig, S2H Fig, S3D Fig) illustrating the need for a detailed investigation of the bacteria-epithelial cell interaction *in vivo* [61].

### Infection-induced innate stimulation and intraepithelial proliferation

**Fig 2:**
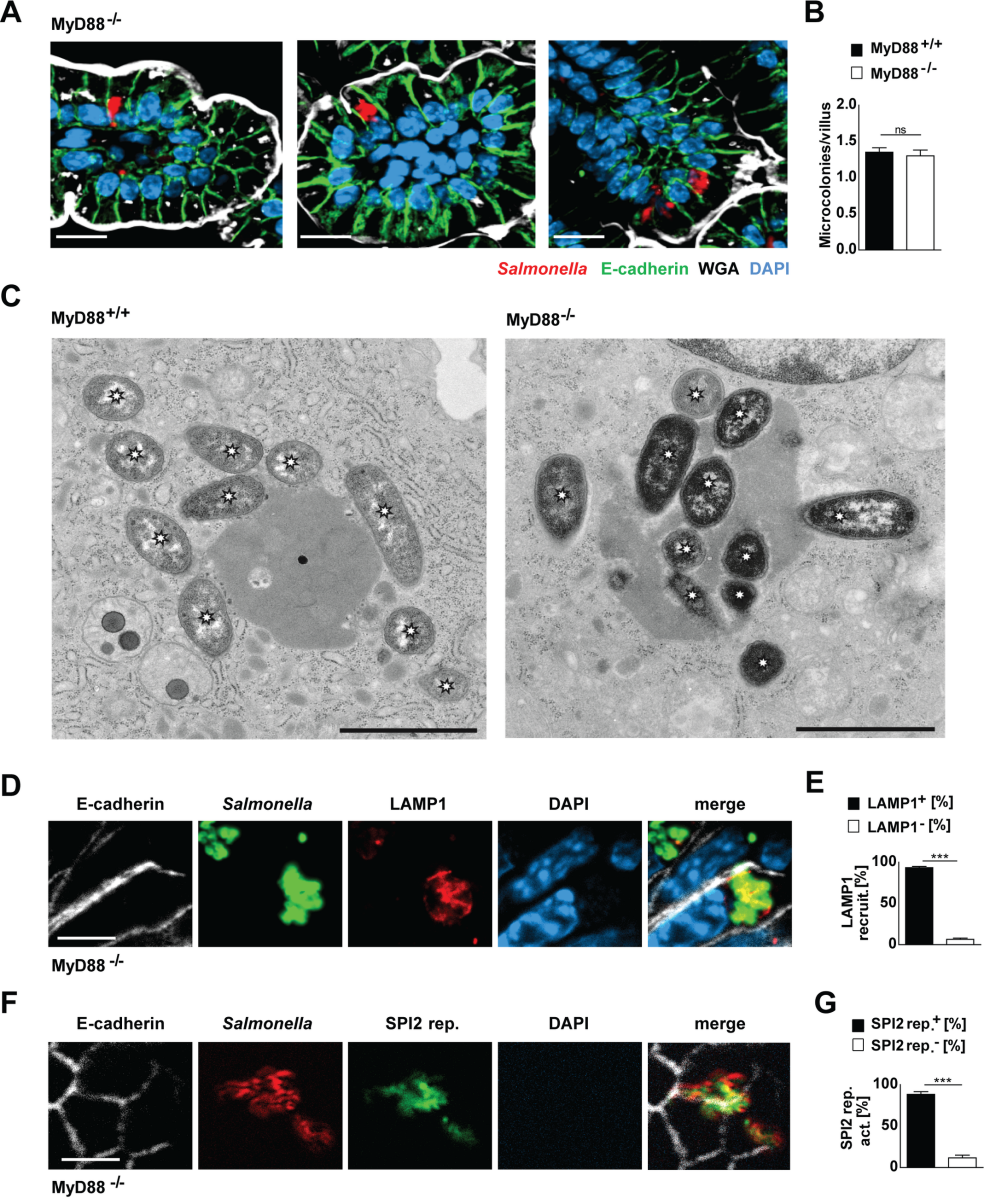
The influence of MyD88-dependent innate immune signaling on intraepithelial microcolony formation. 1-day-old MyD88^+/+^ and MyD88^-/-^ mice were orally infected with 100 CFU *S*. Typhimurium WT. **(A)** 4 days after infection, small intestinal tissues were collected and analyzed by immunostaining. Three representative images showing *S*. Typhimurium (red) forming intraepithelial microcolonies in MyD88^-/-^ mice. Counterstaining with E-cadherin (green), WGA (white) and DAPI (blue). Bar, 10 μm. For wild type controls see Fig 1D. **(B)** Quantitative evaluation of the number of intraepithelial microcolonies per villus in MyD88^+/+^ and MyD88^-/-^ mice at 4 days p.i.. *S*. Typhimurium microcolonies were quantified in 20–30 villi per animal (n = 6–8). Results represent the mean ± SD. **(C)** Transmission electron microscopy (TEM) images of intraepithelial *Salmonella* in MyD88^+/+^ (left panel) and MyD88^-/-^ mice (right panel). Asterisks highlight bacteria. Bar, 2 μm. **(D)** Co-immunostaining for *Salmonella* (green) and LAMP1 (red) in small intestinal tissue sections at day 4 p.i.. Counterstaining with E-cadherin (white) and DAPI (blue). Bar, 5 μm. **(E)** Quantitative evaluation of the percentage of intraepithelial *S*. Typhimurium associated with LAMP1 staining. Four neonates were analyzed at day 4 p.i.. Results represent the mean ± SD. **(F)** Co-immumostaining for *Salmonella* (red) and the GFP expressing SPI2 reporter (pM973, green) in small intestinal tissue sections at day 4 p.i.. Counterstaining with E-cadherin (white) and DAPI (blue). Bar, 5 μm. **(G)** Quantitative analysis of the percentage of intraepithelial *S*. Typhimurium expressing the SPI2 reporter. Microcolonies from tissue sections from four neonates were analyzed at day 4 p.i.. Results represent the mean ± SD.

Host innate immune recognition through Toll-like receptor (TLR)2, 4 and 9 and signaling through the common adapter molecule MyD88 has previously been shown to provide the stimulatory signal for SPI2 effector gene expression and represent a prerequisite for the expression of SPI2-encoded effector genes, intracellular growth and microcolony formation in myeloid cells [60]. Interestingly, innate immune stimulation in *Salmonella*-infected neonate mice was also induced by TLR stimulation and mediated by the common adapter molecule MyD88 [50]. We therefore tested the requirement of intact MyD88-dependent signaling for intraepithelial *Salmonella* proliferation *in vivo*. As expected, MyD88-deficient mice exhibited significantly reduced epithelial *Cxcl2* and *Cxcl5* mRNA expression (S4A and S4B Fig). However, in contrast to the situation in myeloid cells, *Salmonella* maintained its ability to generate intraepithelial microcolonies with similar numbers of microcolonies per villus also in the absence of MyD88 signaling as illustrated by immunostaining and transmission electron microscopy (Fig 2A–2C). Also, LAMP1 was recruited to the majority of *Salmonella*-containing vacuole (SCV) in enterocytes and the SPI2 reporter was induced in the absence of MyD88-dependent signal transduction (Fig 2D–2G). The disease course was significantly accelerated rather than delayed, most probably as a result of an impaired MyD88-mediated antimicrobial host defence (S4C Fig). Thus, intraepithelial *Salmonella* proliferation and microcolony formation occurred independently of MyD88-mediated host innate immune signaling *in vivo*.

### Requirement of SPI1-T3SS effector molecules for intraepithelial microcolony formation

**Fig 3:**
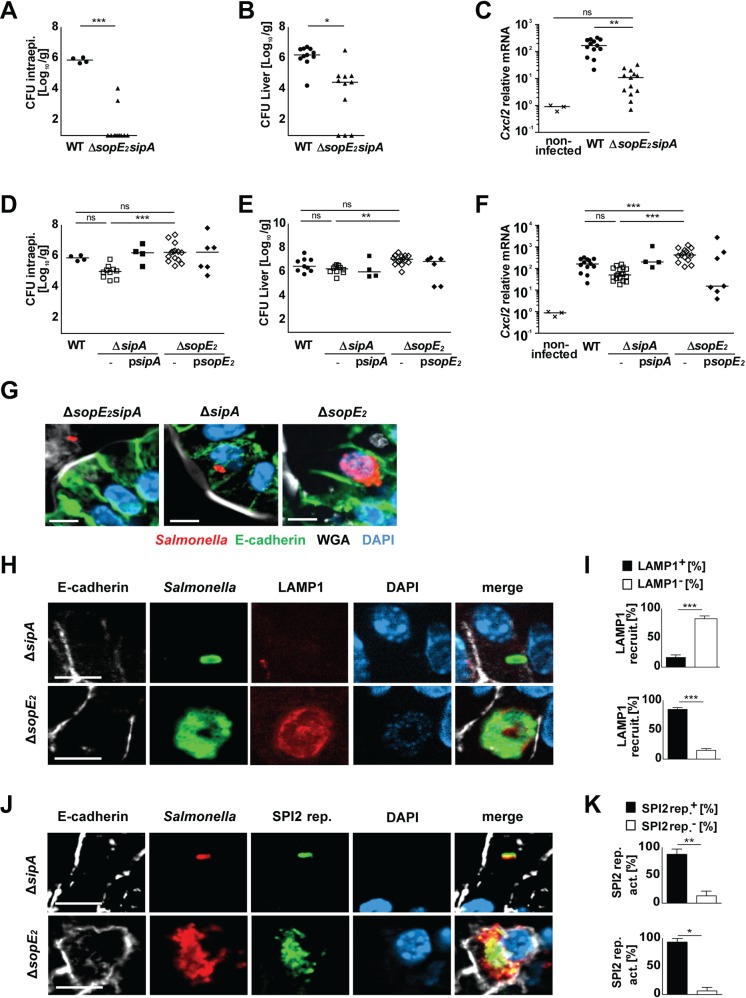
The redundant role of SipA and SopE_2_ for enterocyte invasion. **(A-C)** 1-day-old C57BL/6 mice were orally infected with 100 CFU wild type (WT) (filled circles) or isogenic *sopE*_*2*_*sipA* mutant *S*. Typhimurium (filled triangles). Viable counts in **(A)** isolated gentamicin-treated enterocytes and **(B)** total liver tissue homogenate at 4 days p.i.. **(C)** Quantitative RT-PCR for *Cxcl2* mRNA in total RNA prepared from enterocytes isolated at 4 days p.i.. Values were normalized to uninfected age-matched control animals (crosses). Individual values and the mean from at least two independent experiments are shown (n = 4–6 animals per group). The data for uninfected control animals and WT *Salmonella* infected mice are identical to Fig 1A–1C. **(D-F)** 1-day-old C57BL/6 mice were orally infected with 100 CFU WT (filled circles), isogenic *sipA* mutant (open squares), complemented Δ*sipA* p*sipA* (filled squares), isogenic *sopE*_*2*_ mutant (open diamonds), or complemented Δ*sopE*_*2*_ p*sopE*_*2*_ (filled diamonds) *Salmonella*. Viable counts in **(D)** isolated gentamicin-treated enterocytes and **(E)** total liver tissue homogenate at 4 days p.i.. **(F)** Quantitative RT-PCR for *Cxcl2* mRNA in total RNA prepared from enterocytes isolated at 4 days p.i.. Values were normalized to uninfected age-matched control animals (crosses). Individual values and the mean from at least two independent experiments are shown (n = 3–5 animals per group). The data for uninfected control animals and *Salmonella* WT infected mice are identical to Fig 1A–1C. **(G)** Immunostaining for *S*. Typhimurium (red) in small intestinal tissue sections at 4 days p.i. with 100 CFU Δ*sopE*_*2*_*sipA*, Δ*sipA*, or Δs*opE*_*2*_ *S*. Typhimurium *Salmonella*. Counterstaining with E-cadherin (green), WGA (white) and DAPI (blue). Bar, 5 μm. **(H)** Co-immunostaining for *Salmonella* Δ*sipA*, Δ*sopE*_*2*_ (green) and LAMP1 (red) in small intestinal tissue sections at day 4 p.i.. Counterstaining with E-cadherin (white) and DAPI (blue). Bar, 5 μm. **(I)** Quantitative evaluation of the percentage of intraepithelial *S*. Typhimurium associated with LAMP1 staining. All microcolonies from three tissue sections per infected neonate were analyzed (n = 5) at day 4 p.i.. Results represent the mean ± SD. **(J)** Co-immunostaining for *Salmonella* Δ*sipA* or Δ*sopE*_*2*_ (red) and the GFP-expressing SPI2 reporter (pM973; green) in small intestinal tissue sections at day 4 p.i.. Counterstaining with E-cadherin (white) and DAPI (blue). Bar, 5μm. **(K)** Quantitative analysis of the percentage of intraepithelial *S*. Typhimurium expressing the SPI2 reporter. All microcolonies from three tissue sections per infected neonate were analyzed (n = 3) at day 4 p.i.. Results represent the mean ± SD.

Although SipA or SopE_2_ were sufficient to facilitate enterocyte invasion, *Salmonella* failed to induce intraepithelial microcolony formation. This suggested that intracellular proliferation required the contribution of SPI1-T3SS effector proteins beyond their invasion-promoting function. We first investigated the requirement of SipA and/or SopE_2_ for intraepithelial proliferation. As expected, Δ*sopE*_*2*_*sipA Salmonella* exhibited severely impaired enterocyte invasion, transcriptional stimulation and organ spread (Fig 3A–3C, S5A–S5C Fig). In contrast, *sipA* or *sopE*_*2*_ mutant bacteria displayed only a minor phenotype with intact enterocyte invasion, transcriptional stimulation and spread to systemic organs (Fig 3D–3F, S5D–S5F Fig). Strikingly, however, *sopE*_*2*_ mutant bacteria similar to wild type *Salmonella* generated LAMP1-positive intraepithelial microcolonies, whereas Δ*sipA Salmonella* failed to do so (Fig 3G, 3H and 3I). In contrast, both Δ*sopE*_*2*_ and Δ*sipA Salmonella* exhibited detectable intraepithelial SPI2 reporter activity (Fig 3J and 3K). Consistent with intracellular proliferation of Δ*sopE*_*2*_ *Salmonella*, the presence of SipA as compared to SopE_2_ resulted in significantly higher intraepithelial viable counts (Fig 3D). The enhanced number of intraepithelial bacteria was in turn associated with increased *Cxcl2* mRNA expression as well as augmented spread to spleen, MLN and liver tissue (Fig 3E and 3F, S5D and S5E Fig).

**Fig 4:**
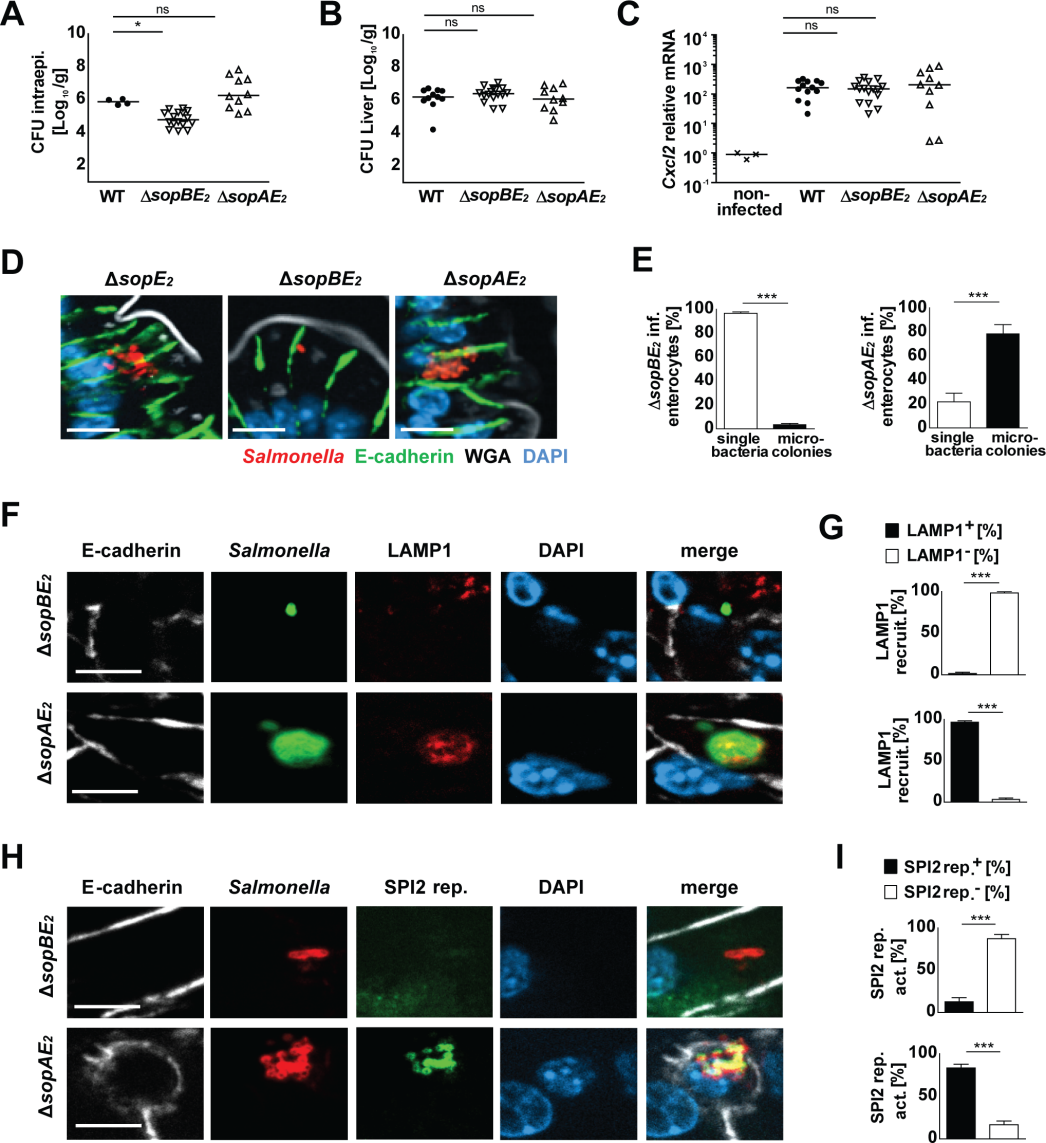
Analysis of *sopBE*_*2*_ and *sopAE*_*2*_ double mutant *S*. Typhimurium. **(A-C)** 1-day-old C57BL/6 mice were orally infected with 100 CFU wild type (WT) (filled circles), isogenic Δ*sopBE*_*2*_ (inverted open triangles), or Δ*sopAE*_*2*_ (open triangles) *S*. Typhimurium. Viable counts in **(A)** isolated gentamicin-treated enterocytes and **(B)** total liver tissue homogenate at 4 days p.i.. **(C)** Quantitative RT-PCR for *Cxcl2* mRNA in total RNA prepared from enterocytes isolated at 4 days p.i.. Values were normalized to uninfected age-matched control animals (crosses). Individual values and the mean from at least two independent experiments are shown (n = 3–6 animals per group). The data for uninfected control animals and *Salmonella* WT infected mice are identical to Fig 1A–1C. **(D)** Immunostaining for *Salmonella* (red) in small intestinal tissue sections at 4 days p.i. with 100 CFU Δ*sopE*_*2*_, Δ*sopBE*_*2*_, or Δ*sopAE*_*2*_ *S*. Typhimurium. Counterstaining with E-cadherin (green), WGA (white) and DAPI (blue). Bar, 5 μm. **(E)** Percentage of epithelial cells positive for single bacteria or microcolonies (>1 intraepithelial bacterium) at 4 days p.i. with Δ*sopBE*_*2*_ or Δ*sopAE*_*2*_ *S*. Typhimurium. 30 *Salmonella-*positive epithelial cells per infected neonate (n = 8–13) were analyzed. Results represent the mean ± SD. **(F)** Co-immunostaining for *Salmonella* Δ*sopBE*_*2*_ and Δ*sopAE*_*2*_ (green) and LAMP1 (red) in small intestinal tissue sections at day 4 p.i.. Counterstaining with E-cadherin (white) and DAPI (blue). Bar, 5 μm. **(G)** Quantitative evaluation of the percentage of intraepithelial Δ*sopBE*_*2*_ and Δ*sopAE*_*2*_ *S*. Typhimurium associated with LAMP1 staining. All microcolonies from three tissue sections per infected neonate were analyzed (n = 3–4) at day 4 p.i.. Results represent the mean ± SD. **(H)** Co-immunostaining for Δ*sopBE*_*2*_ and Δ*sopAE*_*2*_ *Salmonella* (red) and the GFP-expressing SPI2 reporter (pM973; green) in small intestinal tissue sections at day 4 p.i.. Counterstaining with E-cadherin (white) and DAPI (blue). Bar, 5 μm. **(I)** Quantitative analysis of the percentage of intraepithelial *S*. Typhimurium expressing the SPI2 reporter. All microcolonies from three tissue sections per infected neonate were analyzed (n = 3–4) at day 4 p.i.. Results represent the mean ± SD.

We next defined the role of SopA and/or SopB during microcolony formation, analyzing the phenotype of *sopAE*_*2*_ and *sopBE*_*2*_ double mutant *Salmonella in vivo*. Both SipA expressing *sopAE*_*2*_ and *sopBE*_*2*_ mutants gained access to the intracellular compartment of epithelial cells, spread to systemic anatomical sites and stimulated a potent transcriptional response (Fig 4A–4C, S6A–S6C Fig). Interestingly, *sopBE*_*2*_ mutant *Salmonella* failed to generate microcolonies, whereas the majority of *sopAE*_*2*_ mutant-infected epithelial cells exhibited intraepithelial growth (Fig 4D and 4E). Consistently, significantly lower intraepithelial bacterial numbers were found for Δ*sopBE*_*2*_ but not Δ*sopAE*_*2*_ *Salmonella* as compared to wild type bacteria (Fig 4A). Also, *sopAE*_*2*_ mutant but not *sopBE*_*2*_ mutant *Salmonella* recruited LAMP1 (Fig 4F and 4G) and upregulated SPI2 gene expression (Fig 4H and 4I). These results highlighted the role of SipA and SopB for intraepithelial proliferation and excluded SopA as critical SPI1 component for intraepithelial microcolony formation.

### The role of SopB during the interaction of *Salmonella* with the epithelium *in vivo*

**Fig 5:**
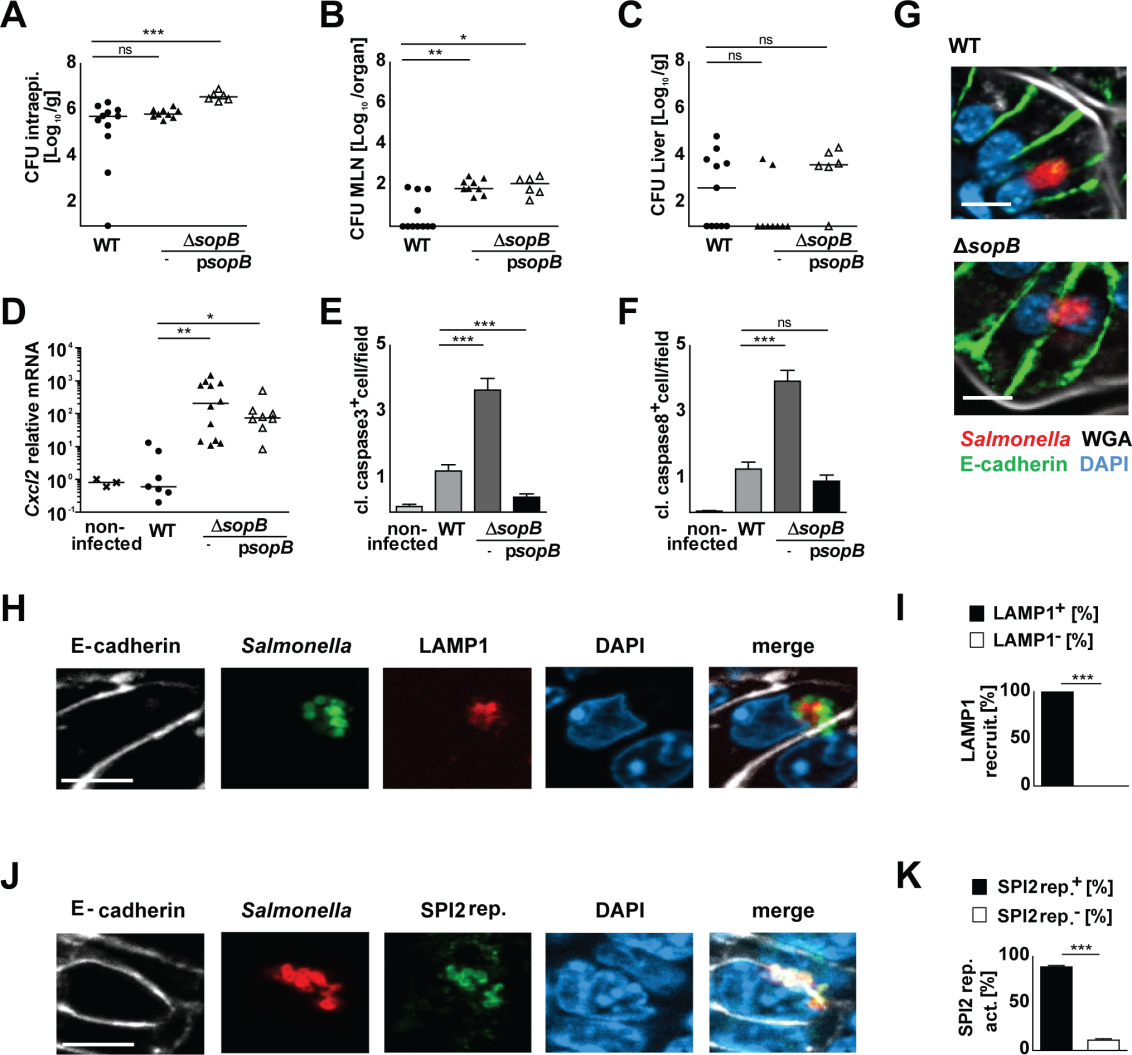
The role of SopB in the interaction between *Salmonella* and the epithelium. 1-day-old C57BL/6 mice were orally infected with 100 CFU wild type (WT) (filled circles), isogenic *sopB* mutant (filled triangles), or p*sopB*-complemented Δ*sopB* (open triangles) *S*. Typhimurium. Viable counts in **(A)** isolated gentamicin-treated enterocytes, **(B)** total MLN and **(C)** total liver tissue homogenate at 2 days p.i.. **(D)** Quantitative RT-PCR for *Cxcl2* mRNA in total RNA prepared from enterocytes isolated at 2 days p.i.. Values were normalized to uninfected age-matched control animals (crosses). Individual values and the mean from at least two independent experiments are shown (n = 3–5 animals per group). **(E)** Quantitative analysis of the number of cleaved caspase 3- and **(F)** cleaved caspase 8 positive cells per 200 times magnification image field. Positive cells from 20 image fields from one section were analyzed per infected neonate (n = 3–6) at day 3 p.i.. Results represent the mean ± SD. **(G)** Immunostaining for *S*. Typhimurium (red) in small intestinal tissue sections at 3 days p.i. with 100 CFU WT and Δ*sopB S*. Typhimurium. Counterstaining with E-cadherin (green), WGA (white) and DAPI (blue). Bar, 5 μm. **(H)** Co-immunostaining for Δ*sopB S*. Typhimurium (green) and LAMP1 (red) in small intestinal tissue sections at day 3 p.i.. Counterstaining with E-cadherin (white) and DAPI (blue). Bar, 5 μm. **(I)** Quantitative evaluation of the percentage of intraepithelial *S*. Typhimurium associated with LAMP1 staining. All microcolonies from three tissue sections per infected neonate were analyzed (n = 3) at day 3 p.i.. Results represent the mean ± SD. **(J)** Co-immumostaining for Δ*sopB S*. Typhimurium (red) and the GFP expressing SPI2 reporter (pM973; green) in small intestinal tissue sections at day 3 p.i.. Counterstaining with E-cadherin (white) and DAPI (blue). Bar, 5 μm. **(K)** Quantitative analysis of the percentage of intraepithelial *S*. Typhimurium expressing the SPI2 reporter. All microcolonies from three tissue sections per infected neonate were analyzed (n = 3) at day 3 p.i.. Results represent the mean ± SD.

To further evaluate SopB as a potential essential effector for intraepithelial proliferation, we next tested single *sopB* mutant *Salmonella in vivo*. Unexpectedly, *sopB* mutant *Salmonella* induced a more severe clinical phenotype with accelerated disease progression. Due to the accelerated disease course induced by Δ*sopB Salmonella*, the following analyses were performed at day 2 and 3 postinfection (p.i.) (Fig 5, S7 Fig). Enterocyte invasion of both wild type and Δ*sopB Salmonella* already occurred at this early time point, but spread to spleen and liver tissue remained low (Fig 5A and 5C, S7A Fig). Infection with Δ*sopB Salmonella* was accompanied by significantly enhanced *Cxcl2* and *Cxcl5* mRNA expression that was not observed during infection with wild type *Salmonella* (Fig 5D, S7B Fig). This increase in epithelial immunostimulation was associated with a significantly accelerated bacterial spread to the MLN (Fig 5B) and enhanced numbers of caspase 3- and caspase 8-positive enterocytes (Fig 5E and 5F, S7C Fig). Since a similar phenotype was not observed after infection with *sopBE*_*2*_ mutant *Salmonella* (Fig 4), these results suggested that SopB by a hitherto unidentified mechanism counteracts the proapoptotic activity induced by SopE_2_ [62, 63].

Strikingly, immunostaining revealed that *Salmonella* deficient solely for *sopB* were still able to proliferate intraepithelially demonstrating that SopB is not essential for intraepithelial microcolony formation (Fig 5G). Consistently, Δ*sopB Salmonella* generated LAMP1-positive intraepithelial endosomal compartments (Fig 5H and 5I) and showed induction of SPI2 reporter gene activity (Fig 5J and 5K). The fact that both *sopE*_*2*_ and *sopB* single mutant *Salmonella* were able to grow intraepithelially (Figs 3G and 5G), whereas *sopBE*_*2*_ double mutant *Salmonella* failed to form intraepithelial microcolonies (Fig 4D and 4E) suggests that SopE_2_ and SopB exert a critical but redundant role during intraepithelial proliferation and microcolony formation.

### The contribution of SipA to mucosal inflammation and intraepithelial proliferation

**Fig 6:**
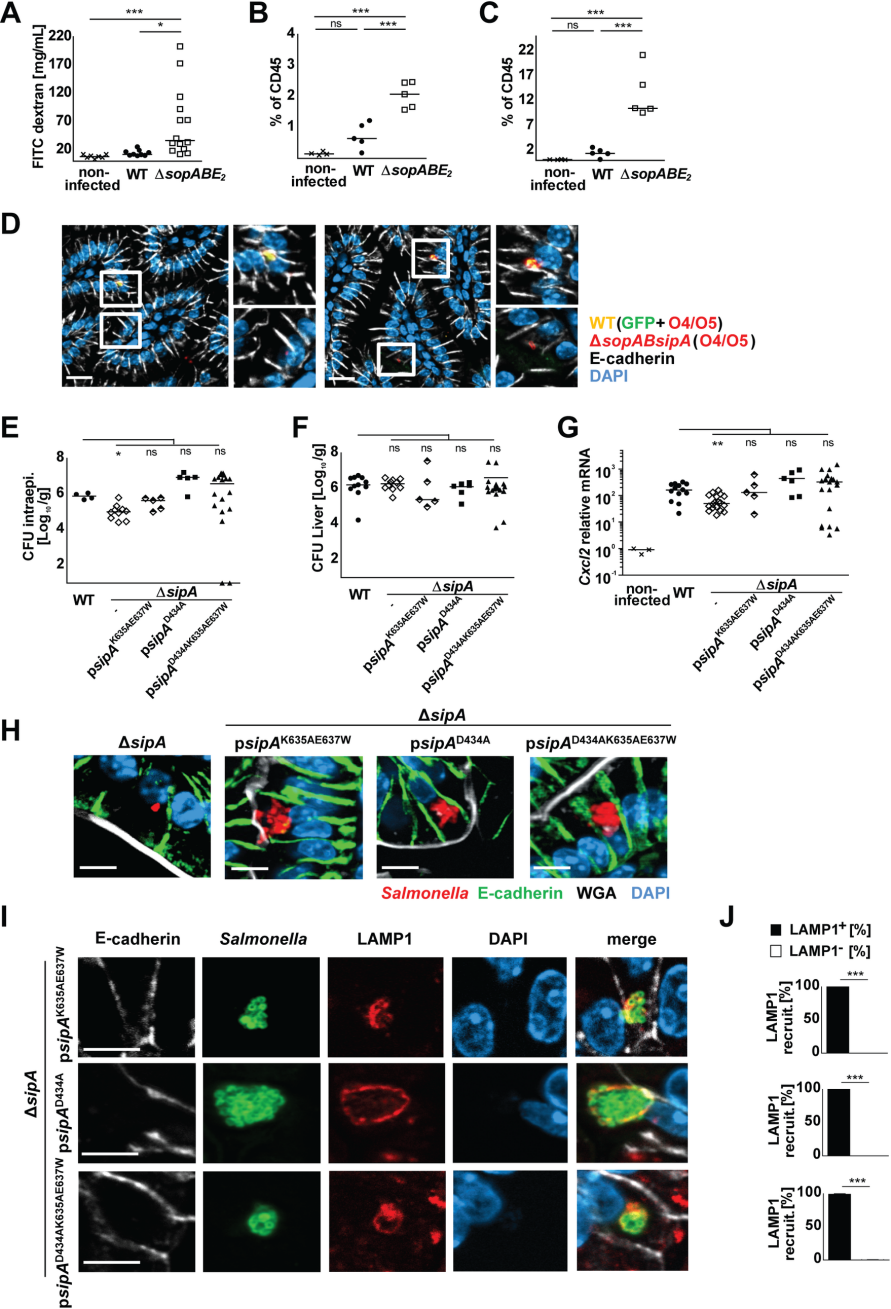
The role of SipA for intraepithelial microcolony formation. **(A)** Mucosal barrier integrity tested by serum quantification 4 hours after oral administration of FITC labeled-4kDa dextran. 1-day-old C57BL/6 mice were left untreated (crosses) or infected with WT (filled circles) or Δ*sopABE*_*2*_ (open squares) *S*. Typhimurium. FITC labeled-4 kDa dextran was quantified in serum at day 3 p.i. as indicated. **(B and C)** Flow cytometric analysis of lamina propria immune cells. 4-day-old mice were left untreated or orally infected with 100 CFU WT or Δ*sopABE*_*2*_ *S*. Typhimurium and total small intestinal leukocytes were analyzed by flow cytometry at day 3 p.i.. **(B)** Monocytes (Ly6C^hi^Ly6G^-^CD11b^+^ MHCII^lo/-^CD45^+^DAPI^-^) and **(C)** neutrophils (Ly6G^+^Ly6C^int^CD11b^+^ MHCII^lo/-^CD45^+^DAPI^-^) are depicted as percentage of CD45^+^ cells in non-infected (crosses), WT (filled circles) and Δ*sopABE*_*2*_ *Salmonella* (open squares). The results represent the mean values from at least two independent experiments (n = 4–6 per group). **(D)** Immunostaining for *Salmonella* in small intestinal tissue sections at 4 days after co-infection with 100 CFU GFP-expressing WT (yellow) and Δ*sopABsipA* (red) *S*. Typhimurium. WT *Salmonella* appear in yellow due to simultaneous staining for O4/O5 antigen (red) and GFP (green). Δ*sopABsipA Salmonella* appear in red due to simultaneous staining for O4/O5 antigen (red). Counterstaining with E-cadherin (white) and DAPI (blue). Bar, 10 μm. **(E-J)** 1-day-old C57BL/6 mice were orally infected with 100 CFU WT (filled circles), Δ*sipA* (open diamonds), Δ*sipA* complemented with p*sipA*^K635A E637W^ (half-filled diamonds), Δ*sipA* complemented with p*sipA*^D434A^ (filled squares), or Δ*sipA* complemented with p*sipA*^D434A K635A E637W^ (filled triangles) *S*. Typhimurium. Viable counts in **(E)** isolated gentamicin-treated enterocytes and **(F)** total liver tissue homogenate at 4 days p.i.. **(G)** Quantitative RT-PCR for *Cxcl2* mRNA in total RNA prepared from enterocytes isolated at 4 days p.i.. Values were normalized to uninfected age-matched control animals (crosses). Individual values and the mean from at least two independent experiments are shown (n = 4–7 animals per group). The data for WT *Salmonella* infected mice and uninfected control animals are identical to Fig 1A–1C. **(H)** Immunostaining for *Salmonella* (red) in small intestinal tissue sections at 4 days p.i. with 100 CFU Δ*sipA*, Δ*sipA* complemented with p*sipA*^K635A E637W^, Δ*sipA* complemented with p*sipA*^D434A^, or Δ*sipA* complemented with p*sipA*^D434A K635A E637W^ *S*. Typhimurium. Counterstaining with E-cadherin (green), WGA (white) and DAPI (blue). Bar, 5 μm. **(I)** Co-immunostaining for LAMP1 (red) and Δ*sipA* complemented with p*sipA*^K635A E637W^, Δ*sipA* complemented with p*sipA*^D434A^, or Δ*sipA* complemented with p*sipA*^D434A K635A E637W^ *S*. Typhimurium (green) in small intestinal tissue sections at day 4 p.i.. Counterstaining with E-cadherin (white) and DAPI (blue). Bar, 5 μm. **(J)** Quantitative evaluation of the percentage of intraepithelial *S*. Typhimurium associated with LAMP1 staining. All microcolonies from three tissue sections per infected neonate were analyzed (n = 3) at day 4 p.i.. Results represent the mean ± SD.

Neonate mice infected with the SipA-expressing *sopABE*_*2*_ triple mutant *Salmonella* exhibited a more severe disease phenotype. Whereas mice infected with wild type *Salmonella* showed low but still significant weight gain during the first days after infection, mice infected with *sopABE*_*2*_ mutant *Salmonella* failed to increase their body weight and exhibited an aggravated course of the disease (S8A and S8B Fig). We therefore infected 4-day-old mice in some experiments to facilitate the analysis. The accelerated disease course of Δ*sopABE*_*2*_ *Salmonella* was associated with an increased tissue inflammation and epithelial barrier impairment illustrated by an enhanced translocation of labelled 4 kDa dextran at day 3 p.i. and a reduced length of the small intestine (Fig 6A, S8C and S8D Fig). Flow cytometric analysis of lamina propria immune cells confirmed a significantly stronger recruitment of monocytes and polymorphonuclear cells (PMN) 3 days after infection with Δ*sopABE*_*2*_ *Salmonella* as compared to wild type *Salmonella* (Fig 6B and 6C, S8E and S8F Fig). Immunohistological detection of infiltrating PMNs corroborated the role of SipA in tissue inflammation. *Salmonella* infection enhanced the number of *lamina propria* PMNs and this effect was significantly reduced after infection with Δ*sipA S*. Typhimurium (S8G and S8H Fig). Notably, genetic complementation with wild type *sipA* (Δ*sipA* p*sipA*) as well as a form of SipA with a point mutation at position 434 (Δ*sipA* p*sipA*^D434A^) previously described to harbor reduced inflammatory activity reversed this phenotype (S8G and S8H Fig). These results demonstrate an intrinsic proinflammatory activity of SipA. They are consistent with previous studies showing that SipA inhibits the phospholipid glutathione peroxidase (GPX4) and leads to enhanced secretion of the proinflammatory chemotactic eicosanoid hepoxillin A_3_ (HXA_3_)[64, 65]. However, our results fail to confirm a significant importance of the caspase 3 cleavage site in SipA for this activity [12, 66].

The proinflammatory activity of SipA was reported to occur independently of enterocyte invasion, following secretion and binding of SipA to the epithelial surface molecule p53-effector related to PMP-22 (PERP) [12, 65, 67, 68]. Consistently, the aggravated disease phenotype was also observed after infection with *Salmonella* that lack SopA, SopB and SopE_2_ and carry two point mutations in SipA (SipA^K635A E637W^) previously shown to impair the actin-modulating activity and reduce the invasion-promoting effect of SipA (S8B Fig). The reduced invasion-promoting activity of SipA^K635A E637W^ as compared to wild type SipA was confirmed by quantification of the number of intraepithelial bacteria 2 days after infection with Δ*sipA S*. Typhimurium complemented with either p*sipA* or p*sipA*^K635A E637W^ (S8I Fig). The invasion-independent activity of SipA suggested that it might act *in trans* and stimulate the epithelium at anatomical locations distant to the site of enterocyte invasion.

To test whether the SipA-mediated immune stimulation contributes to the intraepithelial growth and microcolony formation, we next infected mice with a 1:1 mixture of SipA-sufficient wild type *Salmonella* and SipA-deficient Δ*sopABsipA Salmonella*. As shown in previous experiments (Fig 1A and 1D), Δ*sopABsipA Salmonella* were able to invade epithelial cells through the activity of SopE_2_ but failed to proliferate intraepithelially. Following double infection, wild type *Salmonella* readily formed microcolonies in intestinal epithelial cells (Fig 6D, double stained appearing in yellow). In contrast, Δ*sopABsipA Salmonella* invaded but failed to proliferate intracellularly (Fig 6D, in red). Thus, the induction of a proinflammatory signal by SipA-expressing wild type *Salmonella* was not sufficient to induce a replicative endosomal environment in distant enterocytes and rescue the intraepithelial proliferation defect of SipA-deficient Δ*sopABsipA Salmonella*.

Next, we investigated the intrinsic activity of SipA *in vivo*. We therefore analyzed Δ*sipA Salmonella* complemented *in trans* with the gene for (i) wild type SipA (Δ*sipA* p*sipA*) (ii) SipA with two point mutations at position 635 and 637 previously shown to impair its actin stabilization and reduce the invasion-promoting activity (Δ*sipA* p*sipA*^K635A E637W^) [67] (S8I Fig), (iii) SipA that lacks the caspase 3 cleavage site reported to be critical for the proinflammatory activity [66] (Δ*sipA* p*sipA*^D434A^) and (iv) SipA that lacks both, the actin-stabilizing and proinflammatory activity (Δ*sipA* p*sipA*^D434A K635A E637W^). Expression of these constructs was tested in the presence of SopE_2_ to ensure enterocyte invasion and allow the comparative analysis of the different SipA variants in respect to intraepithelial growth and microcolony formation. As expected, Δ*sipA*, Δ*sipA* p*sipA*^K635A E637W^, Δ*sipA* p*sipA*^D434A^ and Δ*sipA* p*sipA*^D434A K635A E637W^ *Salmonella* were able to invade the epithelium (Fig 6E), cause systemic infection (Fig 6F, S9A and S9B Fig) and induce transcriptional stimulation (Fig 6G, S9C Fig). As observed before, Δ*sipA Salmonella* exhibited significantly reduced intraepithelial bacterial numbers and *Cxcl2* mRNA expression, and failed to proliferate and form intraepithelial microcolonies (Fig 6E, 6G and 6H). Infection with Δ*sipA Salmonella* was also associated with a moderately reduced mortality (S9D Fig). In contrast, Δ*sipA Salmonella* complemented with p*sipA*^K635A E637W^, p*sipA*^D434A^, p*sipA*^D434A K635A E637W^ behaved indistinguishably from wild type *Salmonella* and readily formed LAMP1-positive intraepithelial microcolonies (Fig 6I and 6J). Together, these results demonstrate the critical importance of SipA. However, in the presence of SopE_2_, neither the actin-stabilizing activity nor the reported caspase 3-dependent proinflammatory activity were required for intraepithelial proliferation and microcolony formation.

## Discussion

*Salmonella* expressing SopE, SopE_2_ or SipA but not Δ*sopE*_*2*_*sipA Salmonella* were able to invade the epithelium indicating that the production of SopE, SopE_2_ or SipA is necessary and sufficient to facilitate enterocyte invasion *in vivo*. SopE or SopE_2_ activate the Rho GTPases Rac-1 and Cdc42 or only Cdc42, respectively, leading to actin assembly, membrane ruffling and bacterial internalization [22–24, 54, 56]. SipA with its C-terminal domain stabilizes actin filaments, promoting bacterial invasion in the absence of prominent membrane ruffling [9–12, 42, 69]. Their potent activity was illustrated by the finding that the presence of only one of the two effector molecules facilitated enterocyte invasion at levels indistinguishable from wild type bacteria. Notably, also the ubiquitin E3 ligase SopA was previously shown to contribute to invasion of polarized epithelial cells *in vitro* [15, 16, 42]. Also SopB was shown to promote membrane fission and bacterial invasion [18, 19, 70, 71]. It was suggested that SopB supports SopE-mediated actin filament polymerization by recruiting ARNO (Cytohesin2) to the site of invasion [72]. Our *in vivo* results do not support a significant role for SopA or SopB in enterocyte entry. However, although unlikely, we cannot exclude that enterocyte invasion by strains expressing effectors other than sipA or sopE_2_ occurred but remained undetected due to rapid enterocyte apoptosis or exfoliation as observed in adult mice [29].

Consistent with previous *in vitro* results, enterocyte invasion was not sufficient to allow proliferation and microcolony formation despite the presence of a fully functional SPI2 locus [73]. Δ*sopB*, Δ*sopE*_*2*_ and Δ*sopAE*_*2*_ but not Δ*sipA Salmonella* were able to generate intraepithelial microcolonies assigning SipA a critical non-redundant role for intracellular growth. Δ*sipA Salmonella* failed to recruit LAMP1 to the endosomal epithelial compartment, confirming a recent report on the contribution of the NH_2_-terminal domain of SipA (aa1-458) to endosomal maturation and intracellular replication [74]. SipA was recently also shown to promote intracellular proliferation via interaction with the actin nucleator family member Spire2 [69]. In our study, the SipA activity required for intraepithelial microcolony formation was independent of its actin stabilizing function or proinflammatory activity reported to depend on caspase 3 processing [66, 67]. This might be explained by the recently described cooperative action of SipA with the SPI2 effector SifA to promote phagosome maturation and the generation of a replicative intraepithelial compartment [74]. Interestingly, intraepithelial Δ*sipA Salmonella* exhibited expression of the SPI2 reporter indicating that SPI2 effector expression can occur independently of SipA.

Whereas both Δ*sopE*_*2*_ and Δ*sopB Salmonella* readily proliferated in enterocytes, *sopBE*_*2*_ mutant *Salmonella* were unable to form intraepithelial microcolonies. Consistently, Δ*sopBE*_*2*_ and Δ*sopABE*_*2*_ (in contrast to Δ*sopE*_*2*_ or Δ*sopB*) *Salmonella* failed to recruit LAMP1 to the endosomal compartment and to turn on SPI2 gene expression. Thus, SopB and SopE_2_ contributed in a redundant fashion to the generation of a replicative endosomal compartment in enterocytes. Both, SopE_2_ and SopB activate Rho GTPases and mitogen activated protein kinases (MAPK), which might contribute to endosomal modification [22–24, 72]. Alternatively, SopB was described to directly or indirectly lead to the accumulation of phosphatidylinositol 3-phosphate on the outer leaflet of the SCV, altering the recruitment of host cell endocytic trafficking molecules and thereby preventing SCV-lysosomal fusion [17, 18, 75, 76]. Although SopE_2_ was shown to influence endosomal maturation and intracellular proliferation, it is currently unknown how it could compensate for the lack of SopB [77].

Enterocyte invasion was consistently associated with a transcriptional stimulation of the intestinal epithelium. In contrast, non-invasive Δ*sopABE*_*2*_*sipA Salmonella* failed to evoke a significant epithelial response despite the presence of high numbers of bacteria in the intestinal lumen. Also, the presence of a functional T3SS system in the absence of invasion in *sopAE*_*2*_*sipA*, *sopBE*_*2*_*sipA* or *sopE*_*2*_*sipA* mutant bacteria was unable to induce a significant epithelial transcriptional response. Whereas invasion led to a more than 100-fold increase in *Cxcl2* and *Cxcl5* mRNA expression, intraepithelial proliferation only moderately contributed to this response. Our results therefore suggest that the presence of intraepithelial *Salmonella per se* or, alternatively, downstream events such as stimulation of *lamina propria* immune cells as a consequence of penetration of the epithelial barrier drive epithelial transcriptional stimulation [67]. This is consistent with the previously reported requirement of a functional SPI1 system to evoke PMN transmigration and fluid secretion in calves and the bovine ligated loop model [32–34]. It is also in accordance with the presence of a functional SPI1 locus among all isolates from symptomatic human patients [37, 38].

In addition to the stimulation of pattern recognition receptors the activation of host cell signaling pathways by *Salmonella* virulence factors has been described. For example, SopA was reported to contribute to tissue inflammation by targeting two host E3 ubiquitin ligases, TRIM56 and TRIM65 [13, 15, 78]. Also SopB was shown to indirectly stimulate Rho family GTPases and nuclear responses [56]. However, no significant influence of SopA or SopB on chemokine expression was observed in our study. Instead, our results suggest that SopA together with SopB and/or SopE_2_ may contribute to balance the adverse effects of the inflammatory activity of SipA. Additionally, T3SS-mediated translocation of SipA and SopE_2_ was reported to directly induce host cell activation [23, 79]. SopE-mediated activation of the Rho family of small molecular weight GTPase Cdc42 and activation of p21-activated kinase was reported to induce mitogen-activated protein (MAP) kinase and NF-κB stimulation [23, 80, 81]. However, invasive Δ*sopABsipA* and Δ*sopABE*_*2*_ *Salmonella* (in the absence of SipA and SopE_2_, respectively) still provoked potent transcriptional enterocyte stimulation *in vivo*.

SipA (SipA_294-424_) was additionally shown to induce PMN transmigration and tissue inflammation via PKCα-dependent secretion of the chemotactic eicosanoid hepoxillin A_3_ (HXA_3_) [12, 65, 82]. Notably, this pro-inflammatory activity was demonstrated to be independent of actin filament stabilization and enterocyte invasion and thus differed from the invasion-mediated immune stimulation discussed above [52, 65, 67, 82]. Binding of SipA to the epithelial surface molecule p53-effector related to PMP-22 (PERP) was shown to inhibit the phospholipid glutathione peroxidase (GPX4) [12, 65, 67, 68]. This effect was also observed *in vivo* with major influence on epithelial barrier integrity, immune cell infiltration and the outcome of the disease. Consistent with previous reports, immune stimulation was still observed in *sipA*^K635A E637W^ Δ*sopABE*_*2*_ *Salmonella* despite a reduced invasiveness, supporting the idea that SipA acts extracellularly to stimulate the mucosal immune system [65, 68]. However, no influence of this SipA-mediated immune stimulation was observed on intraepithelial proliferation. The fact that Δ*sopABE*_*2*_ *Salmonella* exhibited a more severe proinflammatory effect as compared to SipA-competent wild type bacteria suggests that SopA, SopB or SopE_2_ might counteract this SipA effect *in vivo*.

Enhanced epithelial apoptosis and increased mortality were noted in the absence of SopB, consistent with a recent report that demonstrated protection from Nlrc4/ASC-mediated apoptosis by SopB *in vitro* [20, 83]. SopB might additionally prevent apoptosis by other mechanisms [21, 84]. Epithelial barrier damage after infection with Δ*sopB Salmonella* explains the higher bacterial load in the mesenteric lymph node. Intriguingly, enhanced disease progression following infection with Δ*sopB Salmonella* was absent using Δ*sipAsopBE*_*2*_ and Δ*sopBE*_*2*_ *Salmonella*. This suggests that the apoptosis-promoting effect is SopE_2_-mediated. Indeed, SopE_2_ has long been described to activate the c-jun NH_2_-terminal kinase (JNK) [23, 80] and JNK signaling is known to drive ROS-induced caspase 3-dependent apoptosis [85]. Alternatively, SopE_2_ was recently shown to activate caspase 1 [63]. Thus, our results identify a previously undescribed role for SopB: to protect from SopE_2_-mediated epithelial cell damage.

Despite the lack of intraepithelial microcolonies, Δ*sopABsipA*, Δ*sopABE*_*2*,_ Δ*sopBE*_*2*_ or Δ*sipA Salmonella* efficiently penetrated the epithelial barrier and spread to systemic organs, reaching levels in spleen and liver tissue comparable to wild type *Salmonella*. This suggests that intraepithelial replication, although considered a hallmark of *Salmonella* pathogenesis, does not represent a prerequisite for efficient penetration of the epithelial barrier. This is consistent with the observation by Müller et al., who described SPI1-T3SS dependent penetration of the colon epithelium in the absence of detectable intraepithelial growth [28]. We even cannot exclude that two different bacterial populations simultaneously drive mucosal translocation and systemic spread. These results indicate that the functional relevance of intraepithelial replication and SCV formation has not been fully established and warrants further investigation.

**Fig 7:**
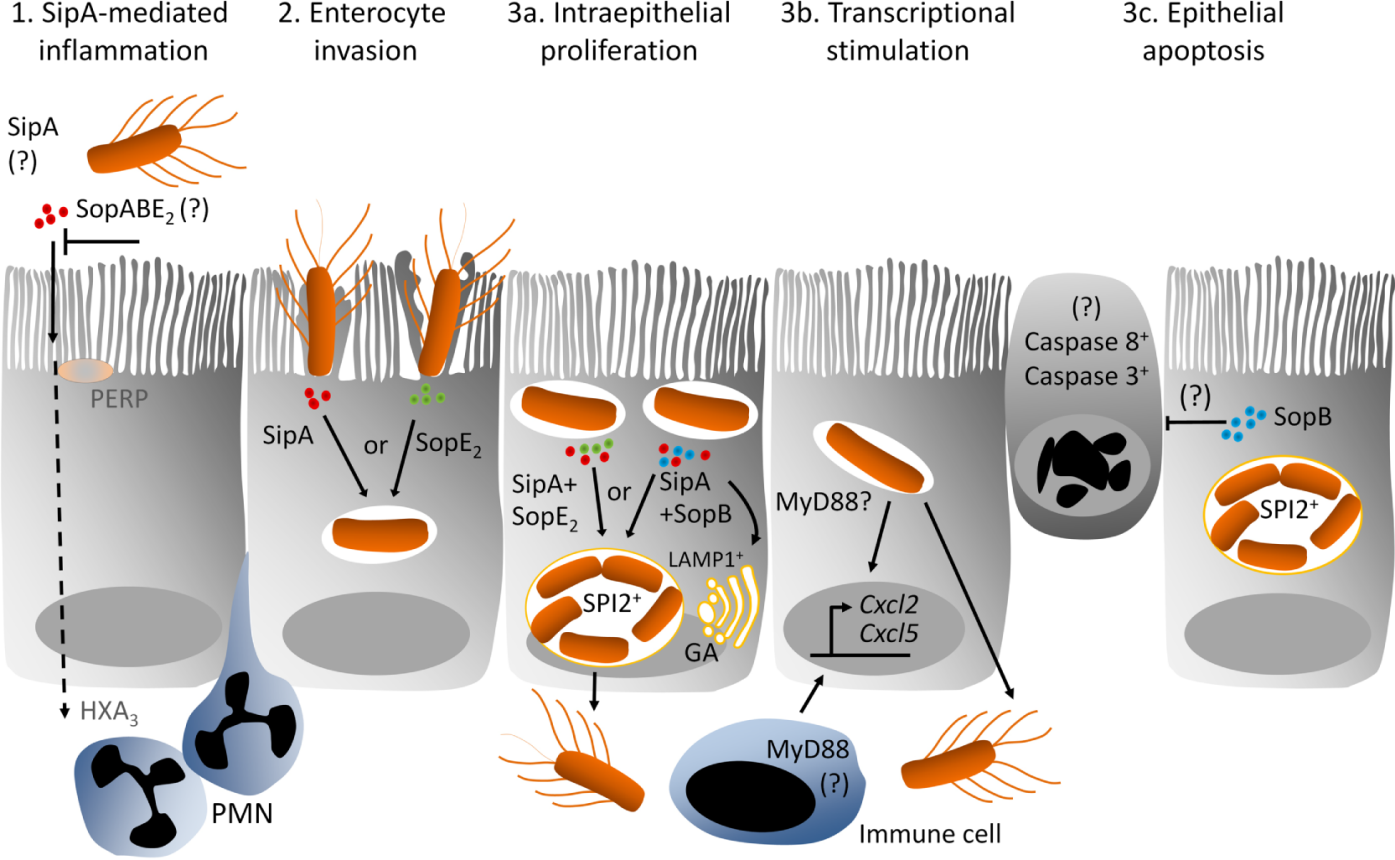
Graphical illustration of the role of the SPI1-T3SS effectors SopA, SopB, SopE_2_ and SipA during enterocyte invasion and intraepithelial proliferation *in vivo*. **(1)** SipA (red dots) promotes the early recruitment of PMNs and causes barrier disruption and disease progression. This has been reported to occur via stimulation of the epithelial surface molecule p53-effector related to PMP-22 (PERP) and activation of the chemotactic eicosanoid hepoxillin A_3_ (HXA_3)_ [68, 89]. This effect appears to be invasion-independent and strongly enhanced in the absence of SopA, SopB and SopE_2_ suggesting that these effectors exert regulatory functions. **(2)** Among the studied effector molecules, expression of SipA (red dots), SopE_2_ (green dots), or SopE (not shown here) alone is sufficient to facilitate enterocyte invasion. Intraepithelial *Salmonella* then reside within a LAMP1 negative endosomal compartment and fail to proliferate or express SPI2 encoded genes. **(3a)** SipA together with SopE_2_ or SipA together with SopB (blue dots) facilitate the recruitment of LAMP1 (yellow membrane) from the Golgi apparatus (GA) and the generation of a replicative compartment with intraepithelial bacterial proliferation and expression of SPI2 effector molecules. **(3b)** Enterocyte invasion or, alternatively, penetration of the epithelial barrier *via* innate stimulation and signaling through MyD88 induce expression of the chemokines *Cxcl2* and *Cxcl5* in the epithelium. **(3c)** SopB appears to directly or indirectly inhibit caspase 3 and caspase 8 mediated epithelial cell apoptosis. GA, golgi apparatus; HXA_3_, hepoxilin A_3_; LAMP1, lysosomal-associated membrane protein 1; PERP, p53-effector related to PMP-22; SPI2, *Salmonella* pathogenicity island 2.

Together, we validate the new neonatal infection model and present the first detailed *in vivo* analysis of the host and bacterial factors required for enterocyte invasion and intraepithelial microcolony formation, hallmark of *Salmonella* pathogenesis (Fig 7, S3 Table). We define the redundant and cooperative function of the SPI1-T3SS effectors SopA, SopB, SopE_2_ and SipA for invasion of the intestinal epithelium and intraepithelial growth and characterize their influence on innate immune stimulation, mucosal translocation and spread to systemic organs. Our results thereby significantly extend our insight in the early steps of the microbial-host interplay *in vivo* and might reveal new targets for future preventive and therapeutic measures.

## Materials and methods

### Bacterial strains and plasmids

All bacterial mutants and plasmids used in this study are listed in the S1 Table. *Salmonella enterica* subsp. *enterica* serovar Typhimurium ATCC14028 were used as wild type bacteria. The *sopABE*_*2*_*sipA* quadruple mutant *S*. Typhimurium carrying the low copy number pWSK29 vector encoding *sopA*, *sopB*, *sipA*, *SopE* or *sopE*_*2*_, or the empty pWSK29 vector as control, as well as the respective isogenic *S*. Typhimurium triple, double or single mutants were used to analyze individual SPI1-T3SS effector molecules. Deletions of genes encoding effector proteins were generated by Red-mediated recombination basically as described before [86]. Strains with multiple deletions of effector genes were generated by P22-mediated transduction of mutant alleles containing the aph or CAT cassette. If required to generate multiple mutations by Red-mediated mutagenesis, antibiotic resistance genes were removed by FLP-mediated recombination between FRT sites. For the generation of a strain with point mutations in chromosomal *sipA* a two-step scarless mutagenesis approach according to Hoffmann et al. was applied [86]. A targeting cassette from pWRG717 was amplified using sipA633 In717 For and sipA939 In717 Rev (S2 Table). *Salmonella* WT harboring pWRG730 was induced for expression of redαβγ and transformed to kanamycin resistance. The resulting sipA mutant allele was transferred to other mutant strains using P22 transduction. The mutant allele sipA K635A E637W was amplified by PCR from plasmid p4758 target strains and the resulted DNA was used to transform MvP2511 haboring pWRG730. Resistance to I-SceI-mediated double strand breaks was used for selection of homologous recombination basically as described by Hoffmann et al. [86]. The point mutations in resulting strain MvP2520 were confirmed by DNA sequencing. Finally, the sopE_2_::aph mutation was introduced by P22 transduction to yield MvP2521.

For complementation of *sipA*, *sopA*, *sopB*, *sopE* or *sopE*_*2*_ deletions, the corresponding genes were amplified from *S*. *enterica* sv. Typhimurium genomic DNA introducing 3’ HA tag sequences for detection of the encoded proteins by immunoblotting. Oligonucleotides for amplification are listed in S2 Table. PCR products were cloned in low copy number vector pWSK29 and *E*. *coli* DH5α was used to propagate plasmids p4041, p4042, p4043 and p4044, SopA::HA, SopB::HA, SopE::HA, and SopE_2_::HA, respectively. Since *sipA* is the terminal gene of the *sicAsipBCDA* operon, the P_*sicA*_ promoter was amplified and cloned upstream of *sipA*::HA to generate p4040. Mutant strains were transformed with complementation plasmids listed in S1 Table. Synthesis and translocation of the effector proteins by *S*. Typhimurium was confirmed by immunolabelling of the HA tag. Furthermore, the plasmids gradually complemented the invasion defect of a multi-effector mutant strain. The function of SipA was additionally analyzed using Δ*sipA Salmonella* carrying the *sipA* gene with point mutations at specific functional positions. Vector p4758 carrying two point mutations in the *sipA* gene at the actin binding site (amino acid position 635 and 637), vector p4890 carrying a point mutation in the *sipA* gene at the caspase 3 motif (amino acid position 434), and vector p4892 carrying all three point mutations in the *sipA* gene were generated by site-directed mutagenesis. Site-directed mutagenesis was performed using the Q5 SDM kit according to the manufacturers’ protocol (NEB). Multiple rounds of mutagenesis were performed if required for the generation of double or triple mutations using primers listed in the S2 Table. The plasmid with constitutive green fluorescent protein (GFP) expression (pGFP, AmpR) was kindly provided by Brendan Cormack, Stanford, USA. The reporter construct pM973 expressing g*fp* under the control of the SPI2 promoter *pssaG* (AmpR) kindly provided by Wolf D. Hardt, ETH Zürich, Switzerland was used to analyze the expression of SPI2 effector molecules by intraepithelial *Salmonella*.

### *In vitro* infection experiments

Intestinal epithelial m-IC_cl2_ cells were cultured as previously described [87]. Bacteria were cultured in Luria Bertani (LB) broth at 37°C in the presence of 100 μg/mL ampicillin, 50 μg/mL kanamycin, or 100 μg/mL carbenicillin. Overnight cultures were diluted 1:10 and incubated at 37°C for approximately 80 min until reaching the logarithmic phase (OD_600_ approximately 0.5). Bacteria were then washed three times in PBS and the OD_600_ was adjusted to 0.55–0.60 corresponding to approximately 2.0×10^8^ CFU bacteria per mL. The bacterial suspension was subsequently diluted to obtain the appropriate infection dose. Bacteria were added to the m-IC_cl2_ cells (2×10^5^ cells per well) at a multiplicity of infection (MOI) of 1:10. The cell culture plate was centrifuged at 300xg for 5 min and incubated at 37°C for 1 h. Cells were washed three times with PBS and cell culture medium supplemented with 100 μg/mL gentamicin (Sigma) was added. After incubation at 37°C for 1 h, cells were washed again three times in PBS and lyzed for 2 min at room temperature in 500 μL 0.1% Triton X-100 (Roth) in aqua dest. Viable bacteria were quantified by serial dilution and plating.

### Ethics statement

All animal experiments were performed in compliance with the German animal protection law (TierSchG) and approved by the local animal welfare committee (Niedersachsische Landesamt für Verbraucherschutz und Lebensmittelsicherheit Oldenburg, Germany; Landesamt für Natur, Umwelt und Verbraucherschutz, North Rhine Westfalia) under the code 8402.04.2015A073, 84–02.042015.A067, 84–02.042015.A065 and 81–02.04.2017.A397 including all approved changes.

### *In vivo* infection experiments

Adult C57BL/6J wild type and B6.129P2(SJL)-*MyD88*^*tm1*.*1Defr*^/J (MyD88^-/-^, stock no. 009088) were obtained from the Jackson Laboratory (Bar Harbour, USA) and bred locally under SPF conditions. Bacteria were cultured as described above. 1- and 4-day-old animals were orally infected with 100 CFU of wild type *S*. Typhimurium or the indicated isogenic mutants in a volume of 1 μL PBS. At various time points post infection (p.i.), liver, mesenteric lymph mode (MLN) and spleen were collected and homogenized in sterile PBS. Viable counts were obtained by serial dilution and plating on LB agar plates supplemented with the appropriate antibiotics. Alternatively, small intestinal tissues were collected at the indicated time point p.i. and prepared for immunostaining or electron microscopy. For co-infection, 100 CFU of *Salmonella* wild type carrying pGFP and *sopABsipA* mutant *Salmonella* were administered orally to 1-day-old mice at a ratio of 1:1 in 2 μl.

### Primary epithelial cell isolation

Primary small intestinal epithelial cells (IEC) from neonate mice were isolated as previously described [87]. Briefly, the small intestinal tissue was cut in small pieces and incubated in 30 mM EDTA/PBS at 37°C for 10 min. IEC were detached from the underlying tissue by shaking. Cells were then filtered through a 100 μm nylon cell strainer (BD Falcon) and harvested by centrifugation at 300xg for 10 min. The cell pellet was resuspended in 10% FCS/PBS and harvested by centrifugation at 300xg for 10 min. To obtain the intraepithelial bacterial count, IEC were treated with 100 μg/mL gentamicin for 1 h at room temperature as previously described and subsequently lyzed and plated in serial dilutions on selective LB agar plates [50].

### Gene expression analysis

Total RNA was extracted from isolated IEC using TRIzol (Invitrogen) and the RNA concentration was determined on a NanoDrop 1000 spectrophotometer (Thermo Scientific). First-strand complementary DNA (cDNA) was synthesized from 5 μg total RNA using Oligo-dT primers, RevertAid reverse transcriptase and RiboLock RNase inhibitor (ThermoFisher Scientific). RT-PCR was performed using the Taqman technology with an absolute QPCR ROX mix (Thermo Scientific), sample cDNA and the Taqman probes *Hprt* (Mm00446968-m1), *Cxcl2* (Mm00436450_m1) and *Cxcl5* (Mm00436451_g1) (Life Technologies). Results were calculated by the Δ2-CT method. For data analysis, values were normalized to the *hprt* housekeeping gene and were presented as fold induction over age-matched controls.

### FITC dextran-mediated analysis of epithelial barrier function

Infected mice were orally administered 2 μL of a 0.6 mg/μL 4kDa FITC dextran solution (TdB Consultancy) at the indicated time point p.i.. After four hours, serum was collected and the serum concentration of FITC dextran was measured by fluorometry using a SpectraMax i3 at an excitation of 492nm (9 nm bandwidth) and an emission of 518nm (15 nm bandwidth) using a serially diluted FITC dextran solution as standard.

### Immunofluorescence staining

4 μm paraformaldehyde-fixed paraffin-embedded tissue sections were deparaffinized in xylene and rehydrated followed by antigen retrieval in 10 mM sodium citrate and a blocking step with 10% normal donkey serum/5% BSA/PBS. Chicken anti-GFP (Abcam), rabbit anti-*Salmonella* O4 antigen (Abcam), rat anti-LAMP1 (Developmental Studies Hybridoma Bank), rabbit anti-cleaved caspase-3 (Cell Signaling), rabbit anti-cleaved caspase-8 (Cell Signaling), rat anti-PMN (Ly6-6B2, SeroTec) and mouse anti-E-cadherin (BD Transduction Laboratories) antibodies as well as the indicated fluorophore-conjugated secondary antibodies (Jackson ImmunoResearch) were used. Fluorescein-conjugated (Vector) or AF647-conjugated (Invitrogen) Wheat Germ Agglutinin (WGA) was used to detect the mucus layer. Slides were subsequently mounted in DAPI mounting medium (Vector) and images were taken using a Zeiss ApoTome.2 system microscope connected to a Axiocam 506 digital camera. Images were formatted using the ZEN 2.3 imaging software.

### Flow cytometry analysis

For immune cell isolation, the intestine was cut longitudinally and incubated 2x15min in 2mM EDTA/HBSS at 37°C to remove the epithelium. The lamina propria was digested in 30μg/ml Liberase/DNAse (Roche) for 45min at 37°C and filtered through a 100μm nylon cell strainer to obtain a single cell suspension. The following antibodies from Biolegend were used for the FACS analysis: CD45-BV510 (30-F11), Ly6C-PerCPCy5.5 (HK1.4), Ly6G-PE (1A8), CD11b-APCCy7 (M1/70), MHCII-AF488 (M5/115.14.2). Data were acquired with a BD FACS Canto II and analyzed with FlowJo X.

### Transmission electron microscopy

1-day-old MyD88^+/+^ and MyD88^-/-^ mice were infected with wild type *S*. Typhimurium and sacrificed at day 4 p.i.. Tissue samples were prepared for ultrastructural analysis as previously described [88]. After embedding, samples were post-fixed with 1% osmium tetroxide and contrasted with 2% uranyl acetate, both for 2 h. Samples were dehydrated with a graded ethanol series, followed by infiltration with epoxy resin and overnight heat polymerization. Thin, 70 nm sections were prepared using an ultramicrotome Ultracut UCT (Leica Microsystems) and contrasted with 0.2% lead citrate. Sections were analyzed with a JEM-1400 TEM microscope (Jeol) and images were recorded with TemCam-F216 camera using EM MENU software (both Tvips).

### Statistical analysis

The One-way ANOVA Kruskal-Wallis test (with Dunn's posttest) and the Mann-Whitney test were employed for statistical analysis of bacterial counts in organ tissue and the comparative transcriptional analysis. Mortality was analyzed by log-rank (Mantel-Cox) test. The GraphPad Prism Software 5.0 was used for statistical evaluation. p values are indicated as follows: ***p<0.001; **p<0.01, and *p<0.05.

## Supporting information

S1 Fig Comparative analysis of the role of SPI1-T3SS effector molecules SopA, SopB, SipA and SopE_2_ by complementation of *sopABE*_*2*_*sipA* quadruple mutant *Salmonella*. 1-day-old C57BL/6 mice were orally infected with 100 CFU wild type (WT) pWSK (filled circles), quadruple mutant Δ*sopABE*_*2*_*sipA* pWSK (open circles), Δ*sopABE*_*2*_*sipA* p*sopA* (inverted open triangles), Δ*sopABE*_*2*_*sipA* p*sopB* (open triangles), Δ*sopABE*_*2*_*sipA* p*sipA* (open squares), or Δ*sopABE*_*2*_*sipA* p*sopE*_*2*_ (open diamonds) *S*. Typhimurium. Viable counts in **(A)** isolated gentamicin-treated enterocytes at 4 days post infection (p.i.).. **(B)** Quantitative RT-PCR for *Cxcl2* and **(C)** *Cxcl5* mRNA in total RNA prepared from enterocytes isolated at 4 days p.i.. Values were normalized to uninfected age-matched control animals (crosses). Viable counts in **(D)** total MLN homogenate, **(E)** total liver tissue homogenate and **(F)** total spleen tissue homogenate at 4 days post infection (p.i.). Individual values and the mean from at least two independent experiments are shown (n = 5–8 animals per group). **(G)** Immunostaining for *Salmonella* (red) in small intestinal tissue sections at 4 days p.i. with 100 CFU WT pWSK, Δ*sopABE*_*2*_*sipA* pWSK, Δ*sopABE*_*2*_*sipA* p*sopA*, Δ*sopABE*_*2*_*sipA* p*sopB*, Δ*sopABE*_*2*_*sipA* p*sipA*, or Δ*sopABE*_*2*_*sipA* p*sopE*_*2*_ *S*. Typhimurium. Counterstaining with E-cadherin (green), WGA (white) and DAPI (blue). Bar, 5 μm. **(H)** A confluent monolayer of polarized murine intestinal epithelial m-IC_cl2_ cells were infected at a multiplicity of infection (MOI) of 1:10 with WT pWSK, SPI1 mutant Δ*inv*C, Δ*sopABE*_*2*_*sipA* pWSK, Δ*sopABE*_*2*_*sipA* p*sopA*, Δ*sopABE*_*2*_*sipA* p*sopB*, Δ*sopABE*_*2*_*sipA* p*sipA*, or Δ*sopABE*_*2*_*sipA* p*sopE*_*2*_ *S*. Typhimurium for 1 h at 37°C. Cells were subsequently treated with 100 μg/mL gentamicin for 1 h at 37°C, washed three times, and lyzed in 0.1% Triton X-100. The number of viable bacteria in cell lysates and inoculi was determined by serial dilution and plating. The number of intracellular, gentamicin-protected bacteria relative to the inoculum is shown (%). Results represent the mean ± SD.
(TIF)

S2 Fig Comparative analysis of the role of SPI1-T3SS effector molecules SopE_2_ and SopE by complementation of *sopABE*_*2*_*sipA* quadruple mutant *Salmonella*. 1-day-old C57BL/6 mice were orally infected with 100 CFU WT pWSK (filled circles), Δ*sopABE*_*2*_*sipA* pWSK (open circles), Δ*sopABE*_*2*_*sipA* p*sopE*_*2*_ (open diamonds), or Δ*sopABE*_*2*_*sipA* p*sopE* (half-filled diamonds) *S*. Typhimurium. Viable counts in **(A)** isolated gentamicin-treated enterocytes at 4 days p.i.. **(B)** Quantitative RT-PCR for *Cxcl2* and **(C)** *Cxcl5* mRNA in total RNA prepared from enterocytes isolated at 4 days p.i.. Values were normalized to uninfected age-matched control animals (crosses). Viable counts in **(D)** total MLN homogenate, **(E)** total liver tissue homogenate, and **(F)** total spleen tissue homogenate at 4 days p.i.. Individual values and the mean from at least two independent experiments are shown (n = 5–8 animals per group). **(G)** Immunostaining for *Salmonella* (red) in small intestinal tissue sections at 4 days p.i. with 100 CFU WT pWSK or Δ*sopABE*_*2*_*sipA* p*sopE S*. Typhimurium. Counterstaining with E-cadherin (green), WGA (white) and DAPI (blue). Bar, 5 μm. **(H)** Gentamicin protection assay (as described under (H)) was performed with WT pWSK, Δ*sopABE*_*2*_*sipA* pWSK, Δ*sopABE*_*2*_*sipA* p*sopE*_*2*_, or Δ*sopABE*_*2*_*sipA* p*sopE*_._ The number of intracellular, gentamicin-protected bacteria relative to the inoculum is shown (%). Results represent the mean ± SD.
(TIF)

S3 Fig Comparative analysis of the role of SPI1-T3SS effector molecules SopA, SopB, SipA, or SopE_2_ using triple mutants. **(A-C)** 1-day-old C57BL/6 mice were orally infected with 100 CFU WT (filled circles), *sopABE*_*2*_*sipA* quadruple mutant (open circles), *sopBE*_*2*_*sipA* (inverted open triangles), *sopAE*_*2*_*sipA* (open triangles), *sopABE*_*2*_ (open squares), or *sopABsipA* mutant (open diamonds) *S*. Typhimurium. Viable counts in **(A)** MLN and **(B)** total spleen tissue homogenate at 4 days post infection (p.i.). **(C)** Quantitative RT-PCR for *Cxcl5* mRNA in total RNA prepared from enterocytes isolated at 4 days p.i.. Values were normalized to uninfected age-matched control animals (crosses). Individual values and the mean from at least two independent experiments are shown (n = 5–8 animals per group). **(D)** Gentamicin protection assay (as described in S1H Fig) was performed using WTpWSK, Δ*inv*C, Δ*sopABE*_*2*_*sipA*, Δ*sopBE*_*2*_*sipA*, Δ*sopAE*_*2*_*sipA*, Δ*sopABE*_*2*_, or Δ*sopABsipA S*. Typhimurium_._ The number of intracellular, gentamicin-protected bacteria relative to the inoculum is shown (%). Results represent the mean ± SD.
(TIF)

S4 Fig The influence of MyD88-dependent innate immune signaling on intraepithelial microcolony formation. **(A and B)** 1-day-old MyD88^+/+^ and MyD88^-/-^ mice were left untreated or orally infected with 100 CFU wild type *S*. Typhimurium. Relative expression of **(A)** *Cxcl2* and **(B)** *Cxcl5* mRNA in total RNA prepared from enterocytes isolated from non-infected and infected MyD88^+/+^ mice as well as non-infected and infected MyD88^-/-^ mice at 4 days p.i. were measured by quantitative RT-PCR. Relative expression from at least two independent experiments are shown (n = 2–6 animals per group). The data for MyD88^+/+^ animals infected with *S*. Typhimurium WT are identical to Fig 1C and S3C Fig. **(C)** Survival following *S*. Typhimurium infection. 1-day-old MyD88^+/+^ (n = 12; solid line) and MyD88^-/-^ (n = 12; broken line) mice were orally infected with 100 CFU WT *S*. Typhimurium (broken line). Animals that had to be euthanized due to a rise in the clinical score were included in the analysis (see material and methods).
(TIF)

S5 Fig The role of SipA and SopE_2_ for systemic spread and innate immune stimulation. **(A-C)** 1-day-old C57BL/6 mice were orally infected with 100 CFU WT (filled circles) or Δ*sopE*_*2*_*sipA S*. Typhimurium (filled triangels). Viable counts in **(A)** MLN and **(B)** total spleen tissue homogenate at 4 days p.i.. **(C)** Quantitative RT-PCR for *Cxcl5* mRNA in total RNA prepared from enterocytes isolated at 4 days p.i.. Values were normalized to uninfected age-matched control animals (crosses). Individual values and the mean from at least two independent experiments are shown (n = 3–5 animals per group). **(D-F)** 1-day-old C57BL/6 mice were orally infected with 100 CFU WT (filled circles) Δ*sipA* (open squares), Δ*sipA* complemented with p*sipA* (filled squares), Δ*sopE*_*2*_ (open diamonds), or Δ*sopE*_*2*_ complemented with p*sopE*_*2*_ (filled diamonds) *S*. Typhimurium. Viable counts in **(D)** MLN and **(E)** total spleen tissue homogenate at 4 days p.i.. **(F)** Quantitative RT-PCR for *Cxcl5* mRNA in total RNA prepared from enterocytes isolated at 4 days p.i.. Values were normalized to uninfected age-matched control animals (crosses). Individual values and the mean from at least two independent experiments are shown (n = 3–7 animals per group). The data for uninfected control animals and *Salmonella* WT infected mice are identical to S3A–S3C Fig.
(TIF)

S6 Fig The role of SopB/SopE_2_ and SopA/SopE_2_ for systemic spread and innate immune stimulation. **(A-C)** 1-day-old C57BL/6 mice were orally infected with 100 CFU WT (filled circles), Δ*sopBE*_*2*_ (inverted open triangles), or Δ*sopAE*_*2*_ (open triangles) *S*. Typhimurium. Viable counts in **(A)** MLN and **(B)** total spleen tissue homogenate at 4 days p.i.. **(C)** Quantitative RT-PCR for *Cxcl5* mRNA in total RNA prepared from enterocytes isolated at 4 days p.i.. Values were normalized to uninfected age-matched control animals (crosses). Individual values and the mean from at least two independent experiments are shown (n = 3–8 animals per group). The data for uninfected control animals and *Salmonella* WT infected mice are identical to S3A–S3C Fig.
(TIF)

S7 Fig The role of SopB for systemic spread and innate immune stimulation. **(A and B)** 1-day-old C57BL/6 mice were orally infected with 100 CFU wild type (WT) (filled circles), Δ*sopB* (filled triangles), or Δ*sopB* p*sopB* (open triangles) *S*. Typhimurium. Viable counts in **(A)** total spleen tissue homogenate at 2 days post infection (p.i.). **(B)** Quantitative RT-PCR for *Cxcl5* mRNA in total RNA prepared from enterocytes isolated at 2 days p.i.. Values were normalized to uninfected age-matched control animals (crosses). Individual values and the mean from at least two independent experiments are shown (n = 3–5 animals per group). **(C)** Immunostaining for cleaved caspase 3 (cl. caspase 3, upper panel, red) and cleaved caspase 8 (cl. caspase 8, lower panel, red) in small intestinal tissue sections from healthy age-matched control animals (non-infected) or at 3 days p.i. with WT, Δ*sopB* and Δ*sopB* p*sopB S*. Typhimurium. Counterstaining with E-cadherin (green), and DAPI (blue). Bar, 50 μm.
(TIF)

S8 Fig SipA exerts a proinflammatory, disease-promoting influence. **(A)** Postnatal body weight gain of healthy and infected animals. 1-day-old C57BL/6 mice were left untreated (solid line) or orally infected with 100 CFU WT (broken line) or Δ*sopABE*_*2*_ *S*. Typhimurium (dotted line). **(B)** Survival following *S*. Typhimurium infection. 1-day-old C57BL/6 mice were orally infected with 100 CFU WT (solid line), Δ*sopABE*_*2*_ (broken line), or *sipA*^*K635A E637W*^ Δ*sopABE*_*2*_ *S*. Typhimurium (broken line). Animals that had to be euthanized due to a rise in the clinical score were included in the analysis (see material and methods). **(C)** Total length (in cm) of the small intestine at 4 days p.i. and of uninfected age-matched control animals. 1-day-old C57BL/6 mice were infected with 100 CFU WT, Δ*sopBE*_*2*_*sipA* (inverted open triangles), Δ*sopAE*_*2*_*sipA* (open triangles) Δ*sopABE*_*2*_ (open squares) and or Δ*sopABsipA* (open diamonds) *S*. Typhimurium. Individual values and the mean from at least two independent experiments are shown (n = 3–5 animals per group). **(D)** Mucosal barrier integrity tested by serum quantification 4 hours after oral administration of FITC labeled-4kDa dextran. 1-day-old C57BL/6 mice were infected with WT (filled circles), Δ*sopABsipA* (open diamonds), or Δ*sopAE*_*2*_*sipA* (open triangles) *S*. Typhimurium. FITC labeled-4 kDa dextran was quantified in serum at day 4 p.i.. **(E and F)** Flow cytometric analysis of lamina propria immune cells. 1-day-old mice were orally infected with 100 CFU WT, Δ*sopAE*_*2*_*sipA*, Δ*sopABsipA*, or Δ*sopABE*_*2*_ *S*. Typhimurium and total SI leukocytes were analyzed by flow cytometry at day 4 p.i.. **(E)** Monocytes (Ly6C^hi^Ly6G^-^CD11b^+^MHCII^lo/-^CD45^+^DAPI^-^) and **(F)** neutrophils (Ly6G^+^Ly6C^int^CD11b^+^MHCII^lo/-^CD45^+^DAPI^-^) are depicted as % of CD45^+^ cells. The results represent the mean values from at least two independent experiments (n = 4–6 per group). **(G and H)** Quantitative analysis **(G)** and immunostaining **(H)** of PMN infiltrating the small intestinal tissue. PMN from 10–20 image fields obtained from neonates infected with wild type (WT), Δ*sipA*, Δ*sipA* p*sipA*, Δ*sipA* p*sipA*^D434A^ *S*. Typhimurium (n = 3–9) and uninfected age-matched control animals (n = 5) were analyzed at day 4 p.i.. Values and mean are shown. Bar, 25 μm. **(I)** Viable counts in isolated gentamicin-treated enterocytes at 2 days p.i. of 1-day-old mice with 100 CFU WT (filled circle), Δ*sopABE*_*2*_ (open squares), or *sipA*^*K635A E637W*^ Δ*sopABE*_*2*_ *S*. Typhimurium (filled squares). Values and mean from at least two independent experiments are shown (n = 4–6 animals per group).
(TIF)

S9 Fig The role of SipA for intraepithelial microcolony formation. **(A-C)** 1-day-old C57BL/6 mice were orally infected with 100 CFU WT (filled circles), Δ*sipA* (open diamonds), Δ*sipA* complemented with p*sipA*^K635A E637W^ (half-filled diamonds), Δ*sipA* complemented with p*sipA*^D434A^ (filled squares), or Δ*sipA* complemented with p*sipA*^D434A K635A E637W^ (filled triangles) *S*. Typhimurium. Viable counts in **(A)** MLN and **(B)** total spleen tissue homogenate at 4 days p.i.. **(C)** Quantitative RT-PCR for *Cxcl5* mRNA in total RNA prepared from enterocytes isolated at 4 days p.i.. Values were normalized to uninfected age-matched control animals (crosses). Individual values and the mean from at least two independent experiments are shown (n = 4–7 animals per group). The data for uninfected control animals and *Salmonella* WT infected mice are identical to S3A–S3C Fig. **(D)** Survival following *S*. Typhimurium infection. 1-day-old C57BL/6 mice were orally infected with 100 CFU WT (solid line), Δ*sipA* (broken line), or Δ*sipA* complemented with *sipA*^K635A E637W^ (broken line) *S*. Typhimurium. Animals that had to be euthanized due to a rise in the clinical score were included in the analysis (see Material and Methods).
(TIF)

S1 Table *Salmonella* enterica serovar Typhimurium strains and plasmids used in this study. Strain name, designation, genotype description and reference are listed for all strains and plasmids used in the study.
(DOCX)

S2 Table Oligonucleotides used in this study. Oligonucleotide name and sequence are listed for all oligonucleotides used in the study.
(DOC)

S3 Table Phenotype of *Salmonella* mutants. Summary of the results obtained in this study using all bacterial mutants indicating the genotype, the time point of the analysis and the phenotype, namely enterocyte invasion, LAMP1 recruitment to the *Salmonella* microcolony, SPI2 T3SS reporter activation, transcriptional stimulation of the intestinal epithelium, intraepithelial proliferation and spread to spleen and liver tissue.
(DOC)
