# Supplementary figures and images for: Minimal SPI1-T3SS effector requirement for Salmonella enterocyte invasion and intracellular proliferation in vivo
Source: PLoS Pathog. 2018 Mar 9;14(3):e1006925. doi: 10.1371/journal.ppat.1006925 (PMC5862521; doi:10.1371/journal.ppat.1006925)

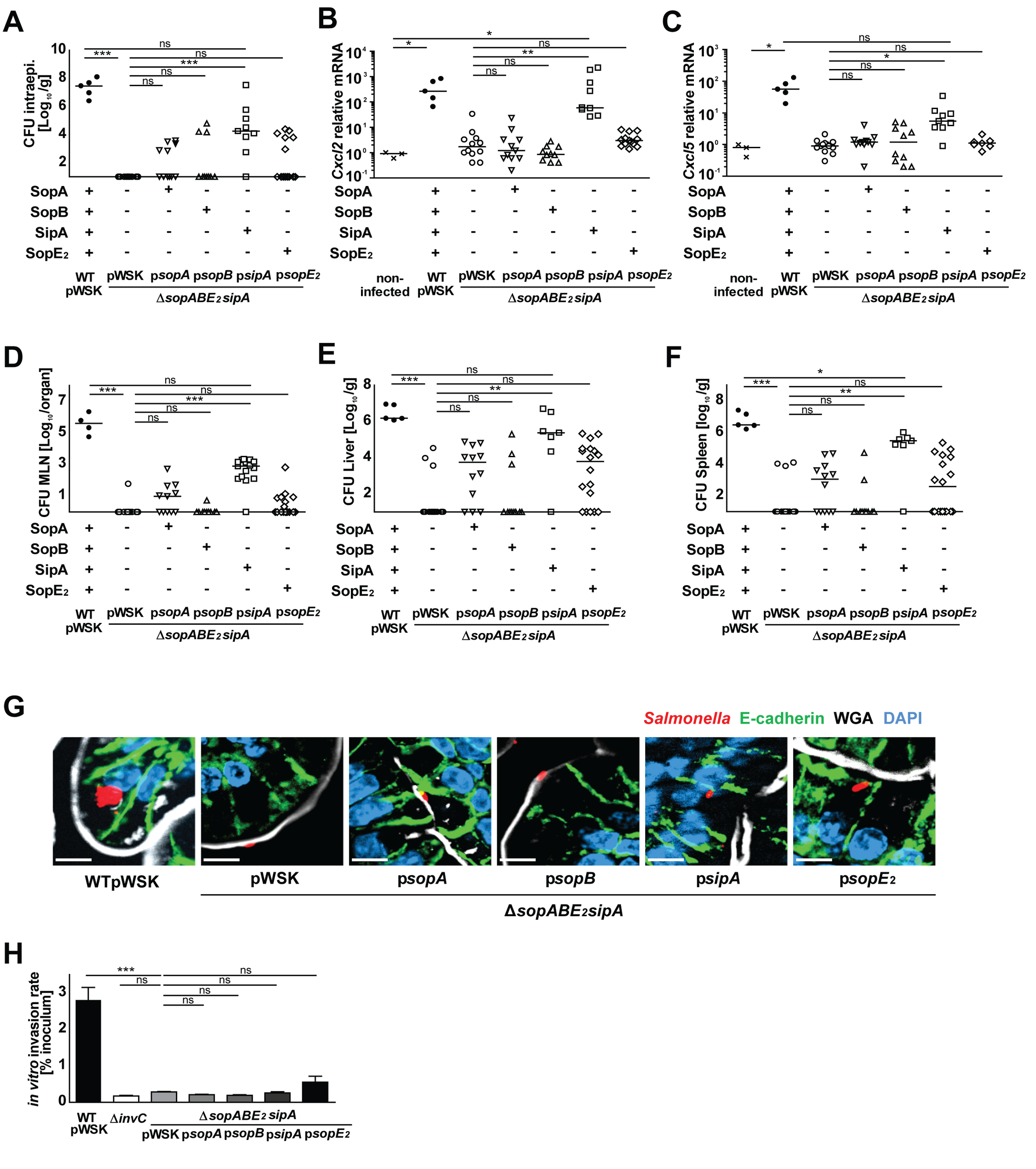

Supplement: S1 Fig — 1-day-old C57BL/6 mice were orally infected with 100 CFU wild type (WT) pWSK (filled circles), quadruple mutant ΔsopABE2sipA pWSK (open circles), ΔsopABE2sipA psopA (inverted open triangles), ΔsopABE2sipA psopB (open triangles), ΔsopABE2sipA psipA (open squares), or ΔsopABE2sipA psopE2 (open diamonds) S. Typhimurium. Viable counts in (A) isolated gentamicin-treated enterocytes at 4 days post infection (p.i.).. (B) Quantitative RT-PCR for Cxcl2 and (C) Cxcl5 mRNA in total RNA prepared from enterocytes isolated at 4 days p.i.. Values were normalized to uninfected age-matched control animals (crosses). Viable counts in (D) total MLN homogenate, (E) total liver tissue homogenate and (F) total spleen tissue homogenate at 4 days post infection (p.i.). Individual values and the mean from at least two independent experiments are shown (n = 5–8 animals per group). (G) Immunostaining for Salmonella (red) in small intestinal tissue sections at 4 days p.i. with 100 CFU WT pWSK, ΔsopABE2sipA pWSK, ΔsopABE2sipA psopA, ΔsopABE2sipA psopB, ΔsopABE2sipA psipA, or ΔsopABE2sipA psopE2 S. Typhimurium. Counterstaining with E-cadherin (green), WGA (white) and DAPI (blue). Bar, 5 μm. (H) A confluent monolayer of polarized murine intestinal epithelial m-ICcl2 cells were infected at a multiplicity of infection (MOI) of 1:10 with WT pWSK, SPI1 mutant ΔinvC, ΔsopABE2sipA pWSK, ΔsopABE2sipA psopA, ΔsopABE2sipA psopB, ΔsopABE2sipA psipA, or ΔsopABE2sipA psopE2 S. Typhimurium for 1 h at 37°C. Cells were subsequently treated with 100 μg/mL gentamicin for 1 h at 37°C, washed three times, and lyzed in 0.1% Triton X-100. The number of viable bacteria in cell lysates and inoculi was determined by serial dilution and plating. The number of intracellular, gentamicin-protected bacteria relative to the inoculum is shown (%). Results represent the mean ± SD. (TIF) [file ppat.1006925.s001.tif]

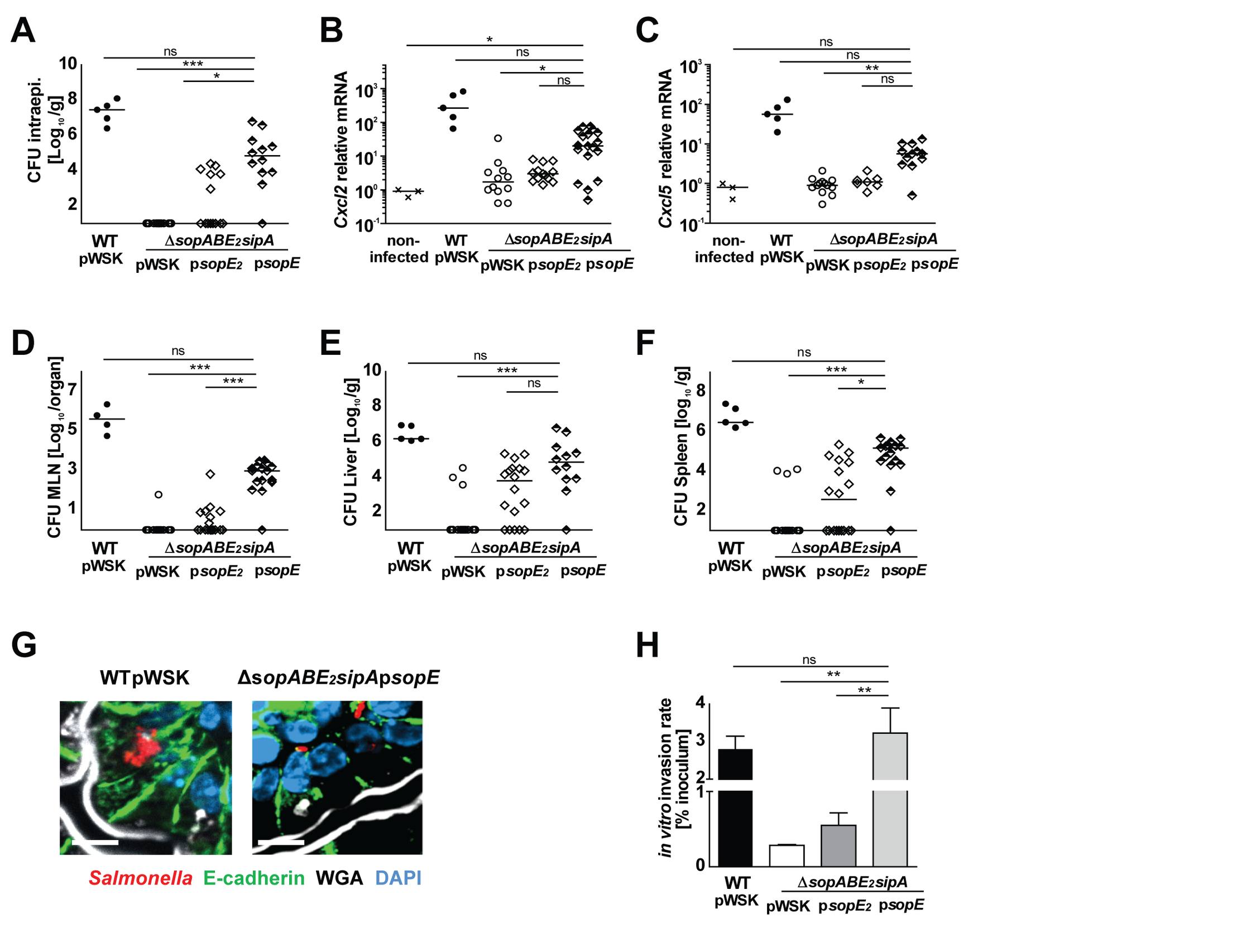

Supplement: S2 Fig — 1-day-old C57BL/6 mice were orally infected with 100 CFU WT pWSK (filled circles), ΔsopABE2sipA pWSK (open circles), ΔsopABE2sipA psopE2 (open diamonds), or ΔsopABE2sipA psopE (half-filled diamonds) S. Typhimurium. Viable counts in (A) isolated gentamicin-treated enterocytes at 4 days p.i.. (B) Quantitative RT-PCR for Cxcl2 and (C) Cxcl5 mRNA in total RNA prepared from enterocytes isolated at 4 days p.i.. Values were normalized to uninfected age-matched control animals (crosses). Viable counts in (D) total MLN homogenate, (E) total liver tissue homogenate, and (F) total spleen tissue homogenate at 4 days p.i.. Individual values and the mean from at least two independent experiments are shown (n = 5–8 animals per group). (G) Immunostaining for Salmonella (red) in small intestinal tissue sections at 4 days p.i. with 100 CFU WT pWSK or ΔsopABE2sipA psopE S. Typhimurium. Counterstaining with E-cadherin (green), WGA (white) and DAPI (blue). Bar, 5 μm. (H) Gentamicin protection assay (as described under (H)) was performed with WT pWSK, ΔsopABE2sipA pWSK, ΔsopABE2sipA psopE2, or ΔsopABE2sipA psopE. The number of intracellular, gentamicin-protected bacteria relative to the inoculum is shown (%). Results represent the mean ± SD. (TIF) [file ppat.1006925.s002.tif]

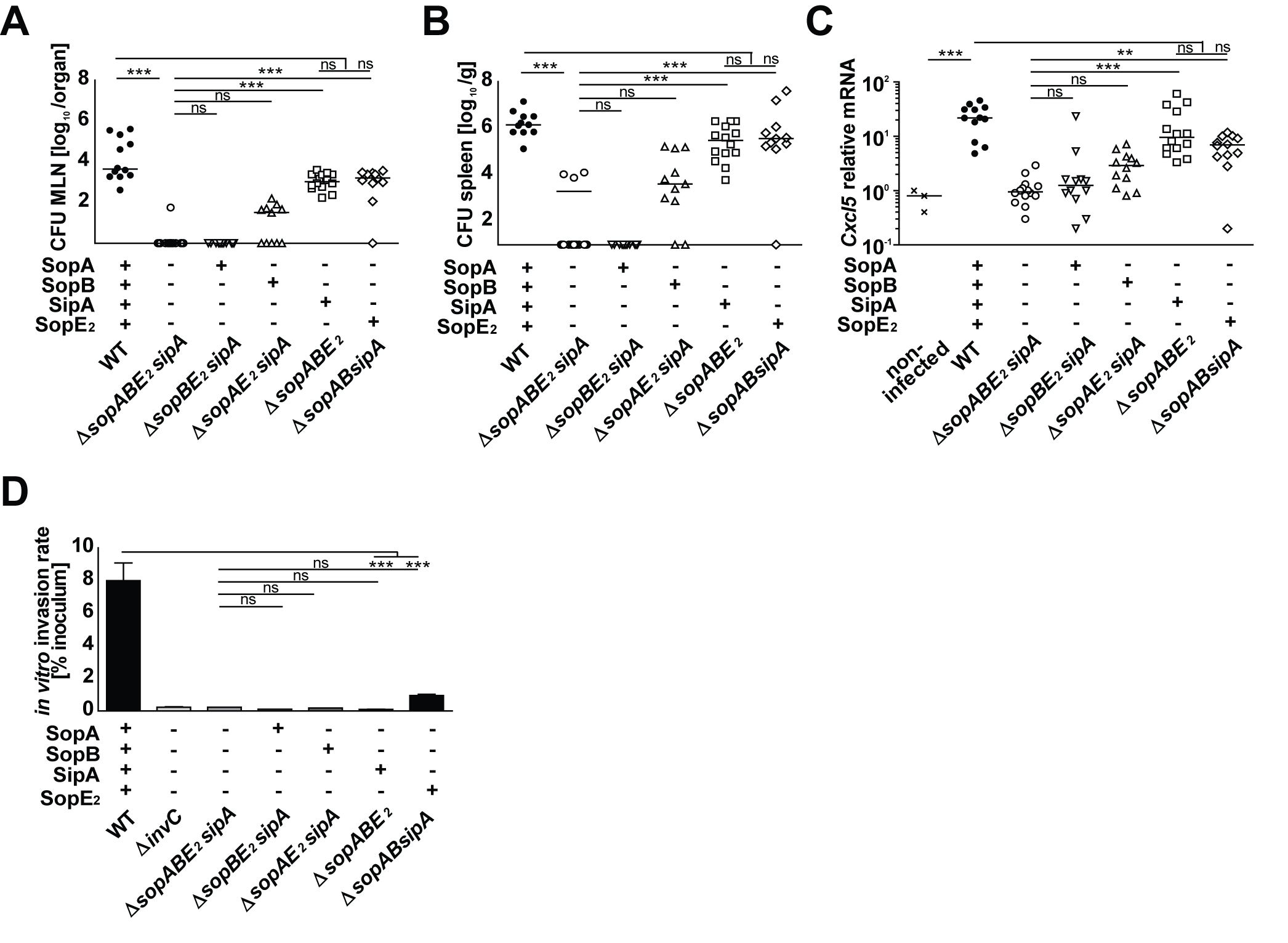

Supplement: S3 Fig — (A-C) 1-day-old C57BL/6 mice were orally infected with 100 CFU WT (filled circles), sopABE2sipA quadruple mutant (open circles), sopBE2sipA (inverted open triangles), sopAE2sipA (open triangles), sopABE2 (open squares), or sopABsipA mutant (open diamonds) S. Typhimurium. Viable counts in (A) MLN and (B) total spleen tissue homogenate at 4 days post infection (p.i.). (C) Quantitative RT-PCR for Cxcl5 mRNA in total RNA prepared from enterocytes isolated at 4 days p.i.. Values were normalized to uninfected age-matched control animals (crosses). Individual values and the mean from at least two independent experiments are shown (n = 5–8 animals per group). (D) Gentamicin protection assay (as described in S1H Fig) was performed using WTpWSK, ΔinvC, ΔsopABE2sipA, ΔsopBE2sipA, ΔsopAE2sipA, ΔsopABE2, or ΔsopABsipA S. Typhimurium. The number of intracellular, gentamicin-protected bacteria relative to the inoculum is shown (%). Results represent the mean ± SD. (TIF) [file ppat.1006925.s003.tif]

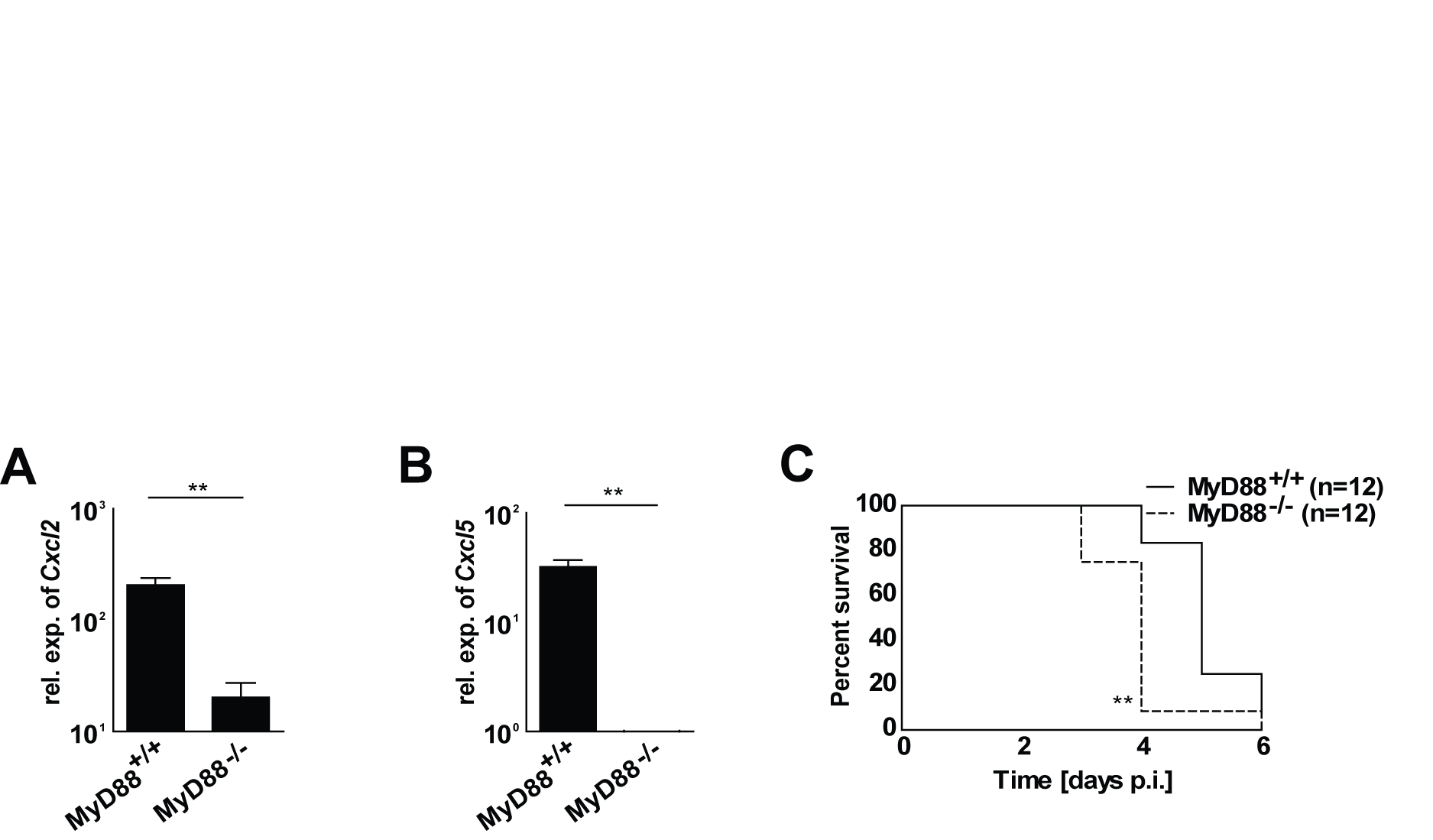

Supplement: S4 Fig — (A and B) 1-day-old MyD88+/+ and MyD88-/- mice were left untreated or orally infected with 100 CFU wild type S. Typhimurium. Relative expression of (A) Cxcl2 and (B) Cxcl5 mRNA in total RNA prepared from enterocytes isolated from non-infected and infected MyD88+/+ mice as well as non-infected and infected MyD88-/- mice at 4 days p.i. were measured by quantitative RT-PCR. Relative expression from at least two independent experiments are shown (n = 2–6 animals per group). The data for MyD88+/+ animals infected with S. Typhimurium WT are identical to Fig 1C and S3C Fig. (C) Survival following S. Typhimurium infection. 1-day-old MyD88+/+ (n = 12; solid line) and MyD88-/- (n = 12; broken line) mice were orally infected with 100 CFU WT S. Typhimurium (broken line). Animals that had to be euthanized due to a rise in the clinical score were included in the analysis (see material and methods). (TIF) [file ppat.1006925.s004.tif]

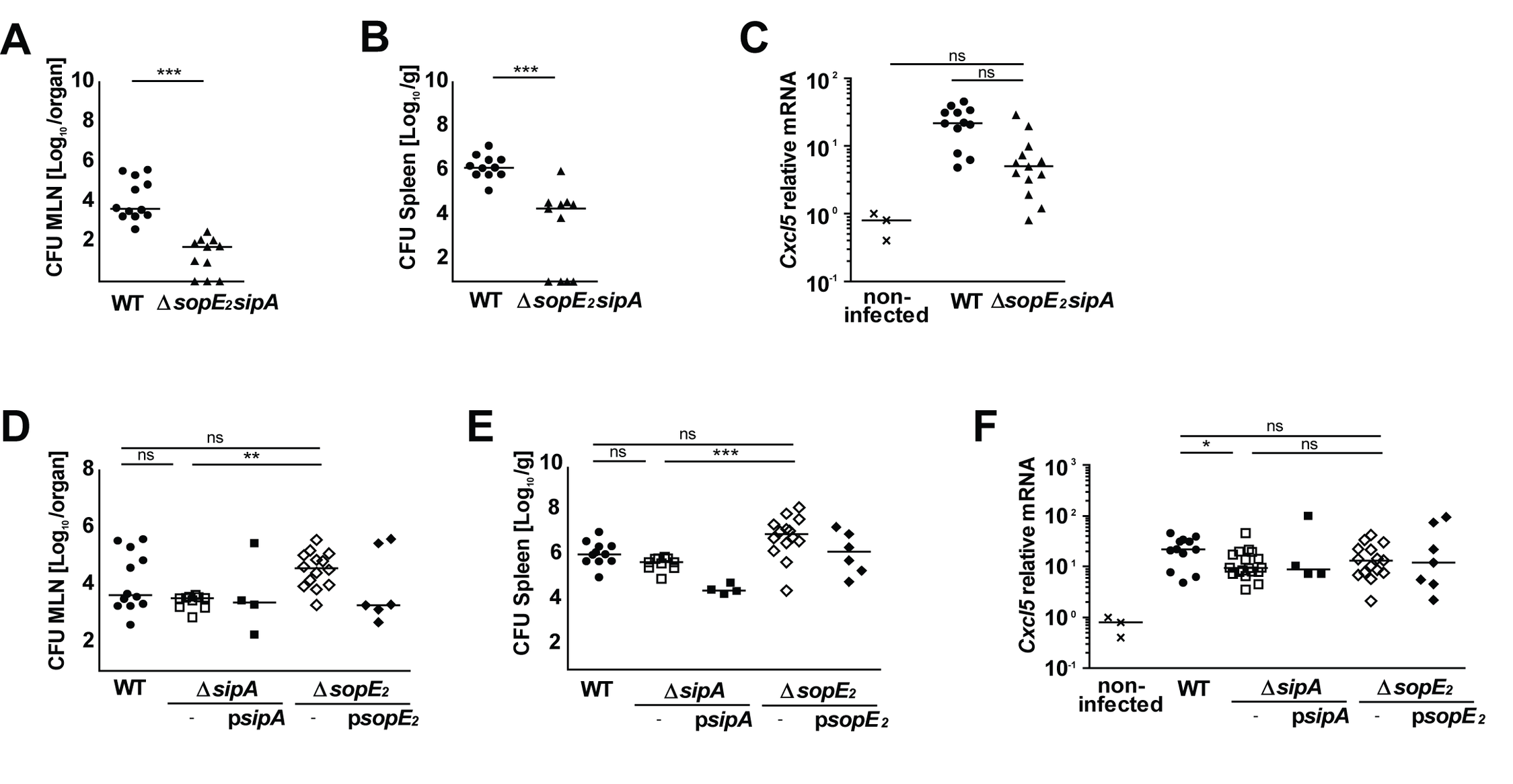

Supplement: S5 Fig — (A-C) 1-day-old C57BL/6 mice were orally infected with 100 CFU WT (filled circles) or ΔsopE2sipA S. Typhimurium (filled triangels). Viable counts in (A) MLN and (B) total spleen tissue homogenate at 4 days p.i.. (C) Quantitative RT-PCR for Cxcl5 mRNA in total RNA prepared from enterocytes isolated at 4 days p.i.. Values were normalized to uninfected age-matched control animals (crosses). Individual values and the mean from at least two independent experiments are shown (n = 3–5 animals per group). (D-F) 1-day-old C57BL/6 mice were orally infected with 100 CFU WT (filled circles) ΔsipA (open squares), ΔsipA complemented with psipA (filled squares), ΔsopE2 (open diamonds), or ΔsopE2 complemented with psopE2 (filled diamonds) S. Typhimurium. Viable counts in (D) MLN and (E) total spleen tissue homogenate at 4 days p.i.. (F) Quantitative RT-PCR for Cxcl5 mRNA in total RNA prepared from enterocytes isolated at 4 days p.i.. Values were normalized to uninfected age-matched control animals (crosses). Individual values and the mean from at least two independent experiments are shown (n = 3–7 animals per group). The data for uninfected control animals and Salmonella WT infected mice are identical to S3A–S3C Fig. (TIF) [file ppat.1006925.s005.tif]

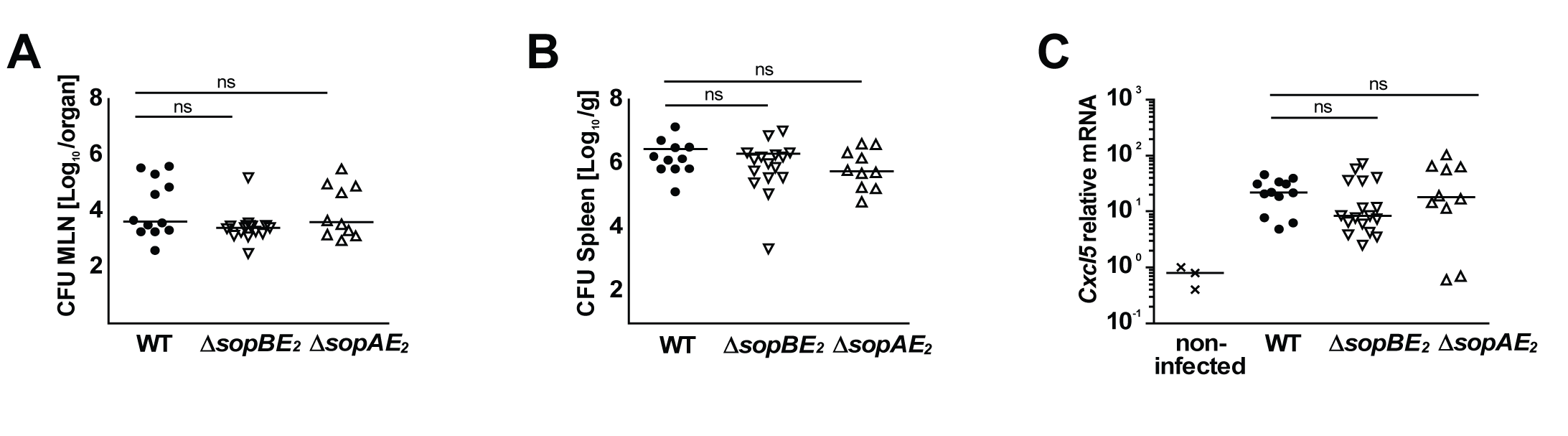

Supplement: S6 Fig — (A-C) 1-day-old C57BL/6 mice were orally infected with 100 CFU WT (filled circles), ΔsopBE2 (inverted open triangles), or ΔsopAE2 (open triangles) S. Typhimurium. Viable counts in (A) MLN and (B) total spleen tissue homogenate at 4 days p.i.. (C) Quantitative RT-PCR for Cxcl5 mRNA in total RNA prepared from enterocytes isolated at 4 days p.i.. Values were normalized to uninfected age-matched control animals (crosses). Individual values and the mean from at least two independent experiments are shown (n = 3–8 animals per group). The data for uninfected control animals and Salmonella WT infected mice are identical to S3A–S3C Fig. (TIF) [file ppat.1006925.s006.tif]

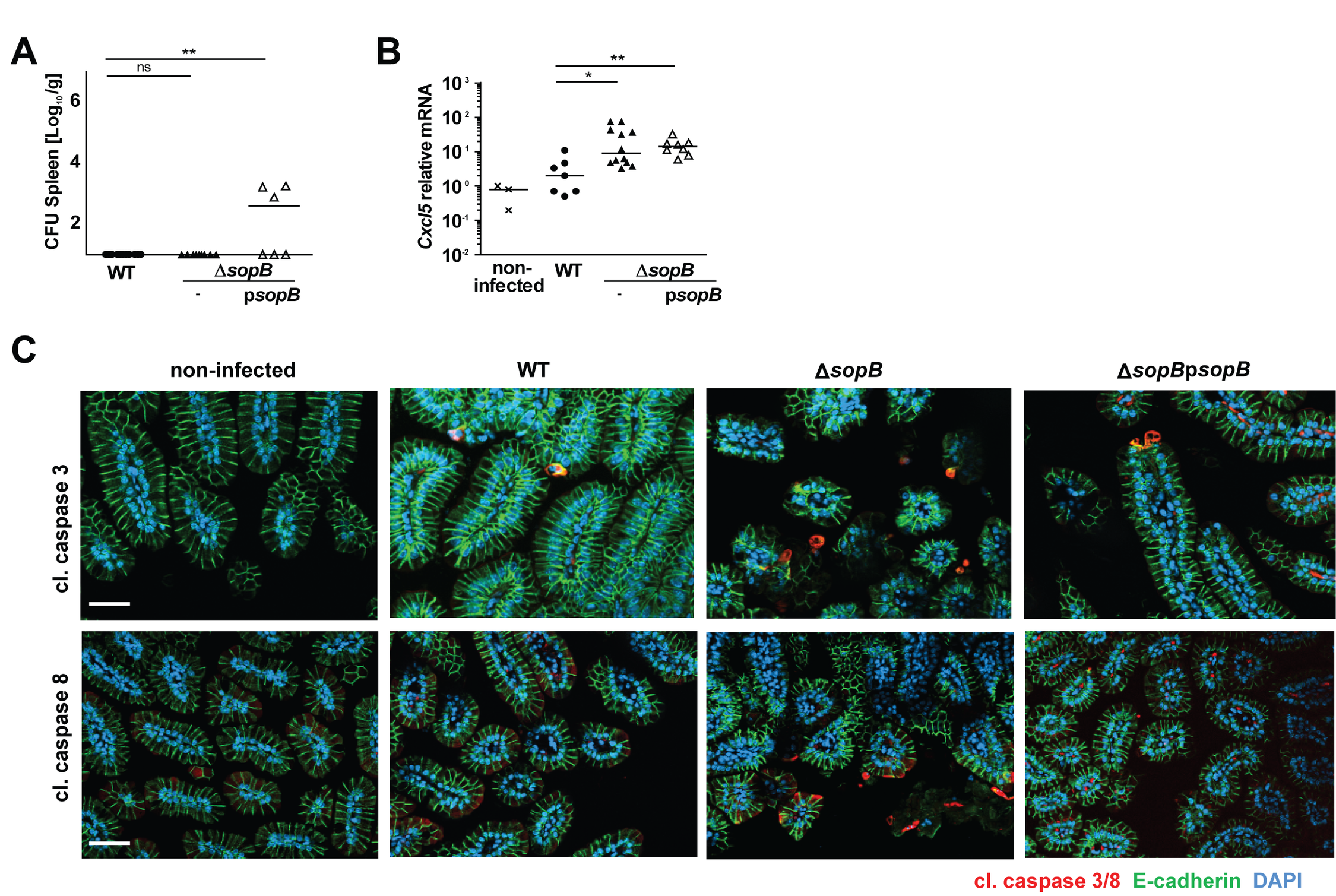

Supplement: S7 Fig — (A and B) 1-day-old C57BL/6 mice were orally infected with 100 CFU wild type (WT) (filled circles), ΔsopB (filled triangles), or ΔsopB psopB (open triangles) S. Typhimurium. Viable counts in (A) total spleen tissue homogenate at 2 days post infection (p.i.). (B) Quantitative RT-PCR for Cxcl5 mRNA in total RNA prepared from enterocytes isolated at 2 days p.i.. Values were normalized to uninfected age-matched control animals (crosses). Individual values and the mean from at least two independent experiments are shown (n = 3–5 animals per group). (C) Immunostaining for cleaved caspase 3 (cl. caspase 3, upper panel, red) and cleaved caspase 8 (cl. caspase 8, lower panel, red) in small intestinal tissue sections from healthy age-matched control animals (non-infected) or at 3 days p.i. with WT, ΔsopB and ΔsopB psopB S. Typhimurium. Counterstaining with E-cadherin (green), and DAPI (blue). Bar, 50 μm. (TIF) [file ppat.1006925.s007.tif]

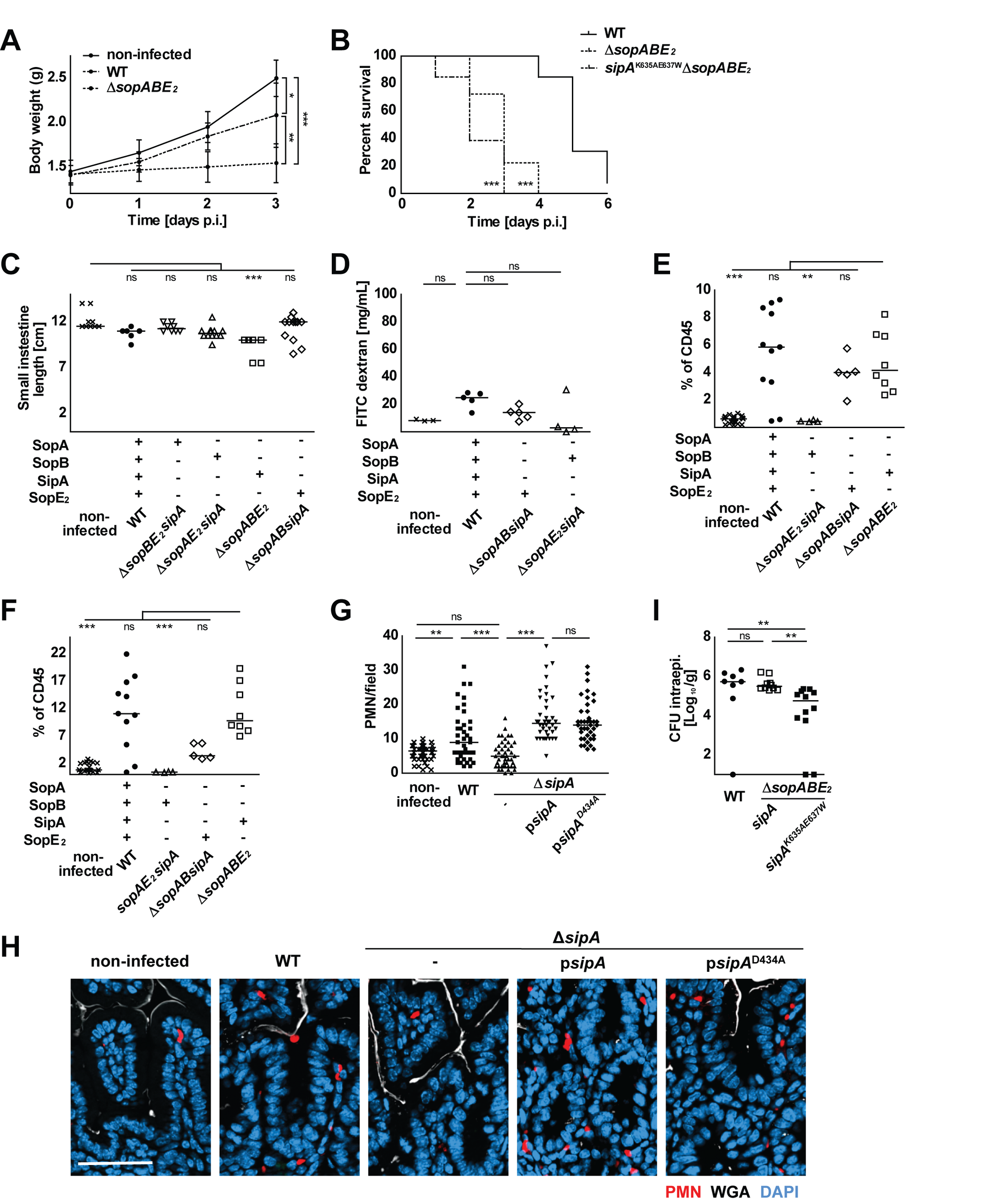

Supplement: S8 Fig — (A) Postnatal body weight gain of healthy and infected animals. 1-day-old C57BL/6 mice were left untreated (solid line) or orally infected with 100 CFU WT (broken line) or ΔsopABE2 S. Typhimurium (dotted line). (B) Survival following S. Typhimurium infection. 1-day-old C57BL/6 mice were orally infected with 100 CFU WT (solid line), ΔsopABE2 (broken line), or sipAK635A E637W ΔsopABE2 S. Typhimurium (broken line). Animals that had to be euthanized due to a rise in the clinical score were included in the analysis (see material and methods). (C) Total length (in cm) of the small intestine at 4 days p.i. and of uninfected age-matched control animals. 1-day-old C57BL/6 mice were infected with 100 CFU WT, ΔsopBE2sipA (inverted open triangles), ΔsopAE2sipA (open triangles) ΔsopABE2 (open squares) and or ΔsopABsipA (open diamonds) S. Typhimurium. Individual values and the mean from at least two independent experiments are shown (n = 3–5 animals per group). (D) Mucosal barrier integrity tested by serum quantification 4 hours after oral administration of FITC labeled-4kDa dextran. 1-day-old C57BL/6 mice were infected with WT (filled circles), ΔsopABsipA (open diamonds), or ΔsopAE2sipA (open triangles) S. Typhimurium. FITC labeled-4 kDa dextran was quantified in serum at day 4 p.i.. (E and F) Flow cytometric analysis of lamina propria immune cells. 1-day-old mice were orally infected with 100 CFU WT, ΔsopAE2sipA, ΔsopABsipA, or ΔsopABE2 S. Typhimurium and total SI leukocytes were analyzed by flow cytometry at day 4 p.i.. (E) Monocytes (Ly6ChiLy6G-CD11b+MHCIIlo/-CD45+DAPI-) and (F) neutrophils (Ly6G+Ly6CintCD11b+MHCIIlo/-CD45+DAPI-) are depicted as % of CD45+ cells. The results represent the mean values from at least two independent experiments (n = 4–6 per group). (G and H) Quantitative analysis (G) and immunostaining (H) of PMN infiltrating the small intestinal tissue. PMN from 10–20 image fields obtained from neonates infected with wild type (WT), ΔsipA, ΔsipA psipA, ΔsipA ps [file ppat.1006925.s008.tif]

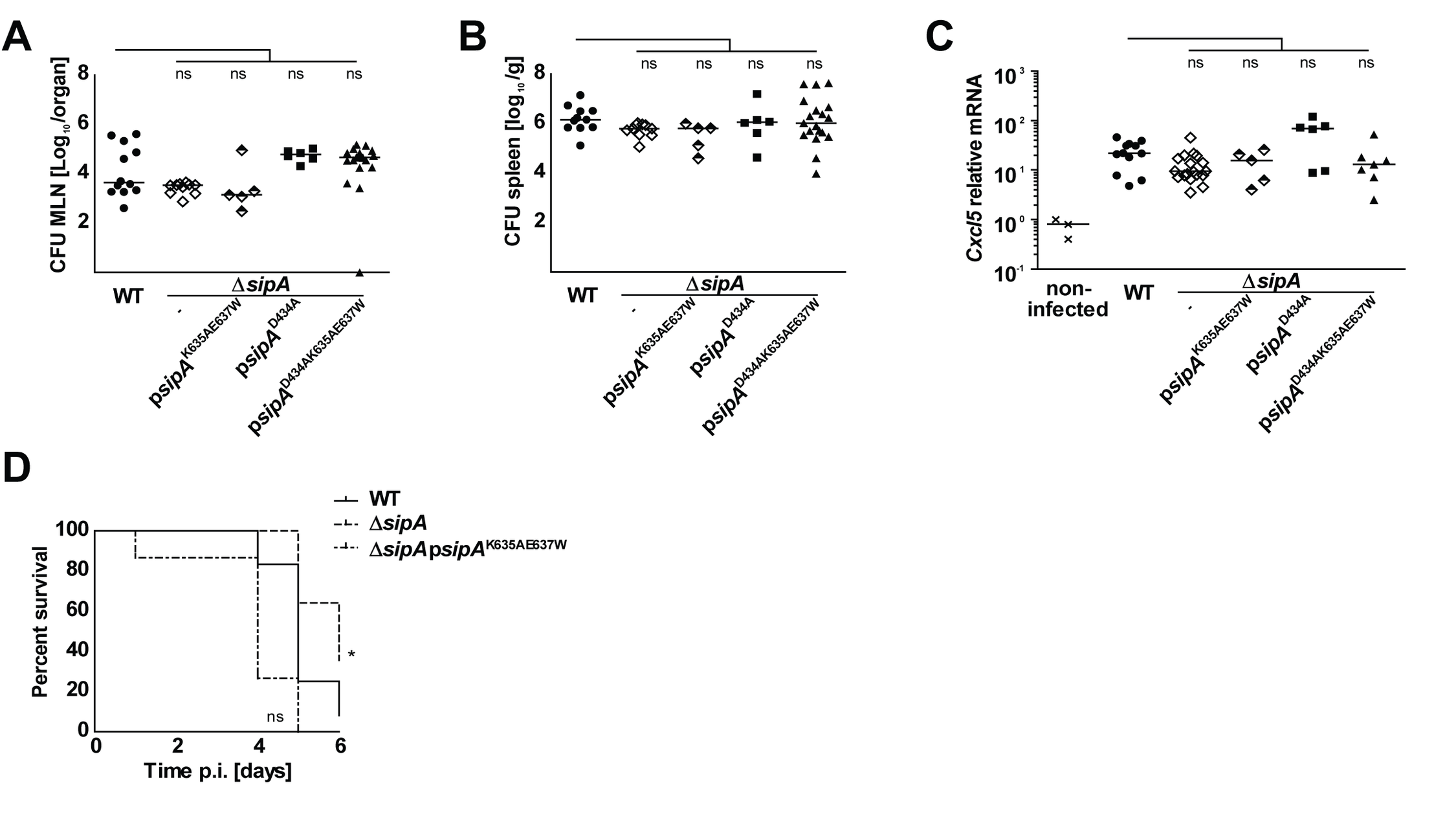

Supplement: S9 Fig — (A-C) 1-day-old C57BL/6 mice were orally infected with 100 CFU WT (filled circles), ΔsipA (open diamonds), ΔsipA complemented with psipAK635A E637W (half-filled diamonds), ΔsipA complemented with psipAD434A (filled squares), or ΔsipA complemented with psipAD434A K635A E637W (filled triangles) S. Typhimurium. Viable counts in (A) MLN and (B) total spleen tissue homogenate at 4 days p.i.. (C) Quantitative RT-PCR for Cxcl5 mRNA in total RNA prepared from enterocytes isolated at 4 days p.i.. Values were normalized to uninfected age-matched control animals (crosses). Individual values and the mean from at least two independent experiments are shown (n = 4–7 animals per group). The data for uninfected control animals and Salmonella WT infected mice are identical to S3A–S3C Fig. (D) Survival following S. Typhimurium infection. 1-day-old C57BL/6 mice were orally infected with 100 CFU WT (solid line), ΔsipA (broken line), or ΔsipA complemented with sipAK635A E637W (broken line) S. Typhimurium. Animals that had to be euthanized due to a rise in the clinical score were included in the analysis (see Material and Methods). (TIF) [file ppat.1006925.s009.tif]
