# Supplementary material for: Minimal SPI1-T3SS effector requirement for Salmonella enterocyte invasion and intracellular proliferation in vivo
Source: PLoS Pathog. 2018 Mar 9;14(3):e1006925. doi: 10.1371/journal.ppat.1006925 (PMC5862521; doi:10.1371/journal.ppat.1006925)
Supplement: S1 Table — Strain name, designation, genotype description and reference are listed for all strains and plasmids used in the study. (DOCX) [file ppat.1006925.s010.docx]

**S1 Table: *Salmonella enterica* serovar Typhimurium strains and plasmids used in this study**

| **Strain name** | **Designation** | **Genotype** | **Reference** |
| --- | --- | --- | --- |
| Wild type | ATCC 14028 | *S*. Typhimurium wild type | NCTC |
| Δ*invC* | MvP813 | Δ*invC* | [1] |
| (construction intermediate) | MvP1880 | Δ*sopA*::FRT Δ*sopB*::FRT | This study |
| Δ*sopABE_2_ sipA* | MvP1891 | Δ*sopB*::FRT Δ*sopA*::FRT Δ*sopE*_2_::FRT Δ*sipA*::FRT | This study |
| Δ*sopBE_2_ sipA* | MvP2016 | Δ*sipA*::FRT Δ*sopB*::*aph* Δ*sopE*_2_::FRT | This study |
| Δ*sopAE_2_ sipA* | MvP2015 | Δ*sipA*::FRT Δ*sopA*::*aph* Δ*sopE*_2_::FRT | This study |
| Δ*sopAB sipA* | MvP2011 | Δ*sipA*::*aph* Δ*sop*A::FRT Δ*sopB*::FRT | This study |
| Δ*sopABE_2_* | MvP1882 | Δ*sopA*::FRT Δ*sopB*::FRT Δ*sopE*_2_::CAT | This study |
| Δ*sopE_2_ sipA* | MvP2307 | Δ*sipA*::FRT Δ*sopE*_2_::FRT | This study |
| Δ*sopAE_2_* | MvP2478 | Δ*sopE*_2_::FRT Δ*sopA*::*aph* | This study |
| Δ*sopBE_2_* | MvP2479 | Δ*sopE*_2_::FRT Δ*sopB*::*aph* | This study |
| Δ*sopE*_2_ | MvP1412 | Δ*sopE*_2_::*aph* | This study |
| Δ*sipA* | MvP1884 | Δ*sipA*::*aph* | This study |
| Δ*sopB* | MvP1208 | Δ*sopB*::*aph* | [2] |
| (construction intermediate) | MvP2501 | I-SceI *sipA*Δ633..639::*aph* | This study |
| (construction intermediate) | MvP2511 | Δ*sopA*::FRT Δ*sopB*::FRT I-SceI *sipA*Δ633..639::*aph* | This study |
| (construction intermediate) | MvP2520 | *sipA* ^K635A E637W^ Δ*sopA*::FRT Δ*sopB*::FRT | This study |
| *sipA* ^K635A E637W^ Δ*sopABE_2_* | MvP2521 | *sipA* ^K635 AE637W^ Δ*sopA*::FRT Δ*sopB*::FRT Δ*sopE*_2_::*aph* | This study |
|  |  |  |  |

**S1 Table (cont.): *Salmonella enterica* serovar Typhimurium strains and plasmids used in this study**

| **Plasmid name** | **Designation** | **Description** | **Reference** |
| --- | --- | --- | --- |
| GFP | pGFP | Expressing green fluorescent protein | Brendan Cormack |
| pWSK29 | pWSK29 | Empty vector | [3] |
| psopA | p4041 | pWSK29 carrying *sopA*::HA | This study |
| psopB | p4042 | pWSK29 carrying *sopB*::HA | This study |
| psipA | p4040 | pWSK29 carrying *sipA*::HA | This study |
| psopE_2_ | p4044 | pWSK29 carrying *sopE_2_*::HA | This study |
| psopE | p4043 | pWSK29 carrying *sopE*::HA | This study |
| pM973 | pM973 | GFP expressed under control of SPI2 promoter p*ssaG* | [4] |
| psipA^K635A E637W^ | p4758 | p4040 carrying *sipA* point mutations K635A E637W | This study |
| psipA^D434A^ | p4890 | p4890 carrying *sipA* point mutation D434A | This study |
| psipA^D434A K635A E637W^ | p4892 | p4892 carrying *sipA* point mutations D434A K635A E637W | This study |

1. Gerlach RG, Claudio N, Rohde M, Jackel D, Wagner C, Hensel M. Cooperation of Salmonella pathogenicity islands 1 and 4 is required to breach epithelial barriers. Cellular microbiology. 2008;10(11):2364-76. doi: 10.1111/j.1462-5822.2008.01218.x. PubMed PMID: 18671822.

2. Rajashekar R, Liebl D, Chikkaballi D, Liss V, Hensel M. Live cell imaging reveals novel functions of Salmonella enterica SPI2-T3SS effector proteins in remodeling of the host cell endosomal system. PloS one. 2014;9(12):e115423. doi: 10.1371/journal.pone.0115423. PubMed PMID: 25522146; PubMed Central PMCID: PMCPMC4270777.

3. Wang RF, Kushner SR. Construction of versatile low-copy-number vectors for cloning, sequencing and gene expression in Escherichia coli. Gene. 1991;100:195-9. PubMed PMID: 2055470.

4. Hapfelmeier S, Stecher B, Barthel M, Kremer M, Muller AJ, Heikenwalder M, et al. The Salmonella pathogenicity island (SPI)-2 and SPI-1 type III secretion systems allow Salmonella serovar typhimurium to trigger colitis via MyD88-dependent and MyD88-independent mechanisms. Journal of immunology. 2005;174(3):1675-85. PubMed PMID: 15661931.

**References**
