# Supplementary material for: Minimal SPI1-T3SS effector requirement for Salmonella enterocyte invasion and intracellular proliferation in vivo
Source: PLoS Pathog. 2018 Mar 9;14(3):e1006925. doi: 10.1371/journal.ppat.1006925 (PMC5862521; doi:10.1371/journal.ppat.1006925)
Supplement: S2 Table — Oligonucleotide name and sequence are listed for all oligonucleotides used in the study. (DOC) [file ppat.1006925.s011.doc]

**S2 Table: Oligonucleotides** used in this study

| **Name** | **Sequence 5’-3’** |
| --- | --- |
| sipA K635A E637W For | gtggACTGACTATGTGTTAAGTAATGTGCTGGACG |
| sipA K635A E637W Rev | cctgcTAGCCTGGTCAGCCCCGC |
| sipA D434A For | TGATGAAGTCgcgGGCGTAACCAG |
| sipA D434A Rev | AAAGAGGTTGTTTCACCC |
| sipA633 In717 For | GATTGCACTGCAGTTTGCCAGAGAGGCGGGGCTGACCAGGAGGGTTTTCCCAGTCACGAC |
| sipA939 In717 Rev | CGTCTCCGATAAGGCCGTCCAGCACATTACTTAACACATATGCTTCCGGCTCGTATGTTG |
| SipA-Del13-For | CTTCCTGCAAGGATAACAGAAGAGGATATTAATAATGGTTATTCCGGGGATCCGTCGACC |
| SipA-Del13-Rev | CTTTTTGTGGATGATGAGTAATGACCTCTTTGAGAGTCTCTGTAGGCTGGAGCTGCTTCG |
| SopE2-Red-Del-For | aaagtgtagctatgcatagttatctaaaaggagaactaccgtgtaggctggagctgcttc |
| SopE2-Red-Del-Rev | taattcatatggttaatagcactattgtatttactaccacatatgaatatcctccttag |
| ProSicA-For-HIII | agcaagcttcaaaacccatcgccgttatg |
| ProSicA-Rev-EcoRI | agtgaattctgtcaccgactttgtagaac |
| SopA-For-XhoI | ttactcgagattttacatttcctgaacacgc |
| SopA-HA-Rev-XbaI | gcttctagattaagcgtagtctgggacgtcgtatgggtacgccccaggccagtggcagg |
| SopB-For-XhoI | ttactcgagtcacggtcttacttgtccggg |
| SopB-HA-Rev-XbaI | gcttctagattaagcgtagtctgggacgtcgtatgggtaagatgtgattaatgaagaaat |
| SopE-For-XhoI | taactcgagtacgctcaacgatcagctcac |
| SopE-HA-Rev-XbaI | gcttctagattaagcgtagtctgggacgtcgtatgggtagggagtgttttgtatatattt |
| SopE2-For-XhoI | taactcgagtacgctcaacgatcagctcac |
| SopE2-HA-Rev-XbaI | gcttctagattaagcgtagtctgggacgtcgTatgggtaggaggcattctgaagatactt |
| SipA-For-EcoRI | tgtgaattcctgcaaggataacagaagagg |
| SipA-HA-Rev-XbaI | gcttctagattaagcgtagtctgggacgtcgattgggtaacgctgcatgtgcaagccatc |
