# Supplementary material for: Minimal SPI1-T3SS effector requirement for Salmonella enterocyte invasion and intracellular proliferation in vivo
Source: PLoS Pathog. 2018 Mar 9;14(3):e1006925. doi: 10.1371/journal.ppat.1006925 (PMC5862521; doi:10.1371/journal.ppat.1006925)
Supplement: S3 Table — Summary of the results obtained in this study using all bacterial mutants indicating the genotype, the time point of the analysis and the phenotype, namely enterocyte invasion, LAMP1 recruitment to the Salmonella microcolony, SPI2 T3SS reporter activation, transcriptional stimulation of the intestinal epithelium, intraepithelial proliferation and spread to spleen and liver tissue. (DOC) [file ppat.1006925.s012.doc]

**S3 Table: Phenotype of *Salmonella* mutants**

| **Genotype** | **Time p.i. [days]** | **Phenotype** | | | | | |
| --- | --- | --- | --- | --- | --- | --- | --- |
|  |  | Enterocytes invasion | LAMP1  recruit. | SPI2 rep.  activation | Transcript. stimulation | Intracellular proliferation | Organ  spread |
| Wild type | 4 | Yes | Yes | Yes | Yes | Yes | Yes |
| Δ*sopABE_2_sipA*pWSK29 |  | No | n.t. | n.t. | No | No | No |
| Δ*sopABE_2_sipA*p*sopA* |  | No | n.t. | n.t. | No | No | No |
| Δ*sopABE_2_sipA*p*sopB* |  | No | n.t. | n.t. | No | No | No |
| Δ*sopABE_2_sipA*p*sipA* |  | Yes | n.t. | n.t. | Yes | No | Yes |
| Δ*sopABE_2_sipA*p*sopE_2_* |  | Yes | n.t. | n.t. | No | No | No |
| Δ*sopABE_2_sipA*p*sopE* |  | Yes | n.t. | n.t. | Yes | No | Yes |
| Δ*sopBE_2_sipA* |  | No | n.t. | n.t. | No | No | No |
| Δ*sopAE_2_sipA* |  | No | n.t. | n.t. | No | No | No |
| Δ*sopABsipA* |  | Yes | No | No | Yes | No | Yes |
| Δ*sopABE_2_* |  | Yes | No | No | Yes | No | Yes |
| Δ*sopE_2_sipA* |  | No | n.t. | n.t. | No | No | No |
| Δ*sopAsopE_2_* |  | Yes | Yes | Yes | Yes | Yes | Yes |
| Δ*sopBsopE_2_* |  | Yes | No | No | Yes | No | Yes |
| Δ*sopE*_2_ |  | Yes | Yes | Yes | Yes | Yes | Yes |
| Δ*sopE*_2_p*sopE_2_* |  | Yes | n.t. | n.t. | Yes | n.t. | Yes |
| Δ*sipA* |  | Yes | No | Yes | Yes | No | Yes |
| Δ*sipA*p*sipA* |  | Yes | n.t. | n.t. | Yes | n.t. | Yes |
| Δ*sipA*p*sipA*^K635AE637W^ |  | Yes | Yes | n.t. | Yes | Yes | Yes |
| Δ*sipA*p*sipA*^D434A^ |  | Yes | Yes | n.t. | Yes | Yes | Yes |
| Δ*sipA*p*sipA*^D434AK635AE637W^ |  | Yes | Yes | n.t. | Yes | Yes | Yes |
| Δ*sopB* | 2/3 | Yes | Yes | Yes | Yes | Yes | No |
| Δ*sopB*p*sopB* |  | Yes | n.t. | n.t. | Yes | n.t. | Yes |

n.t.: not tested
